# Bond topology of chain, ribbon and tube silicates. Part II. Geometrical analysis of infinite 1D arrangements of (*T*O_4_)^*n*−^ tetrahedra

**DOI:** 10.1107/S2053273324002432

**Published:** 2024-04-29

**Authors:** Maxwell Christopher Day, Frank Christopher Hawthorne, Ali Rostami

**Affiliations:** a Department of Earth Sciences, University of Manitoba, Winnipeg, Manitoba R3T 2N2, Canada; b Computer Science Department, Friedrich Schiller University Jena, Jena, 07743, Germany; Universidad del País Vasco, Spain

**Keywords:** bond topology, [*T*O_4_]^*n*−^ tetrahedra, chains of tetrahedra, chain graph, graph embedding, Euclidean space

## Abstract

In Part I of this series, all topologically possible 1-periodic infinite graphs (chain graphs) representing chains of tetrahedra with up to 6–8 vertices (tetrahedra) per repeat unit were generated. This paper examines possible restraints on embedding these chain graphs into Euclidean space such that they are compatible with the metrics of chains of tetrahedra in observed crystal structures. Chain-silicate minerals with *T* = Si^4+^ (plus P^5+^, V^5+^, As^5+^, Al^3+^, Fe^3+^, B^3+^, Be^2+^, Zn^2+^ and Mg^2+^) have a grand nearest-neighbour 〈*T*–*T*〉 distance of 3.06±0.15 Å and a minimum *T*⋯*T* separation of 3.71 Å between non-nearest-neighbour tetrahedra, and in order for embedded chain graphs (called unit-distance graphs) to be possible atomic arrangements in crystals, they must conform to these metrics, a process termed *equalization*. It is shown that equalization of all *acyclic* chain graphs is possible in 2D and 3D, and that equalization of most *cyclic* chain graphs is possible in 3D but not necessarily in 2D. All unique ways in which non-isomorphic vertices may be moved are designated *modes of geometric modification*. If a mode (*m*) is applied to an equalized unit-distance graph such that a new geometrically distinct unit-distance graph is produced without changing the lengths of any edges, the mode is designated as valid (*m*_v_); if a new geometrically distinct unit-distance graph cannot be produced, the mode is invalid (*m*_i_). The parameters *m*_v_ and *m*_i_ are used to define ranges of rigidity of the unit-distance graphs, and are related to the edge-to-vertex ratio, *e*/*n*, of the parent chain graph. The program *GraphT*–*T* was developed to embed any chain graph into Euclidean space subject to the metric restraints on *T*–*T* and *T*⋯*T*. Embedding a selection of chain graphs with differing *e*/*n* ratios shows that the principal reason why many topologically possible chains cannot occur in crystal structures is due to violation of the requirement that *T*⋯*T* > 3.71 Å. Such a restraint becomes increasingly restrictive as *e*/*n* increases and indicates why chains with stoichiometry *T*O_<2.5_ do not occur in crystal structures.

## Abstract 2

It is shown that all possible topologically distinct chain graphs of tetrahedra may be embedded into 3D Euclidean space. In minerals, separations between linked *T* cations are 3.06 (15) Å and between unlinked *T* cations are >3.71 Å, and these distances constrain the ability of embedded chain graphs to occur as structural entities in crystals. Software (*GraphT-T*) allows this embedding to be tested for stereochemical viability.

## 1. Introduction

The intent of our work is to provide a framework for understanding the atomic-scale factors controlling composition, structural variability and occurrence of silicate minerals *sensu lato* in the crust and mantle of the Earth. We have developed a structure hierarchy (Hawthorne, 2014 ▸) for chain-silicate minerals (Day & Hawthorne, 2020 ▸) in which (*T*O_4_)^*n*−^ groups (*T* = Si^4+^ + P^5+^, V^5+^, As^5+^, Al^3+^, Fe^3+^, B^3+^, Be^2+^, Zn^2+^ and Mg^2+^) polymerize infinitely in 1D, and have described their connectivities with translationally symmetric 1-periodic graphs (termed *chain graphs*).

Day & Hawthorne (2022 ▸) derived all possible 1D infinite chain graphs (with up to 6–8 tetrahedra per unit cell) including those that correspond to chains of tetrahedra observed in chain-silicate minerals and related synthetic compounds and those that do not. The number of symmetrically independent vertices (*i.e.* tetrahedra) in the topological unit cell was restricted to 6–8 by Day & Hawthorne (2022 ▸) for two reasons: (i) derivation of all possible non-isomorphic graphs with >6–8 vertices (regardless of vertex connectivity) required unrealistically long computation times (weeks to months), and (ii) chain arrangements with >8 tetrahedra in the topological unit cell are observed in 12 structure types representing 14 minerals, those with <8 tetrahedra in the topological unit cell are observed in 100 structure types representing 406 minerals.

Here, our principal intent is to examine the factors that affect the embedding of these chain graphs generated by Day & Hawthorne (2022 ▸) into 2D and 3D Euclidean space to produce geometric chain graphs (*i.e.* unit-distance graphs) with geometrical characteristics that are compatible with the metrics of crystal structures (realistic interatomic distances). We also wish to understand the topological characteristics of chain graphs and the geometrical characteristics of unit-distance graphs that give rise to different classes of mineral structures: (i) common minerals of high abundance; (ii) common minerals of low abundance; (iii) rare minerals of high abundance; (iv) rare minerals of low abundance; (v) no mineral structures at all. In addition to the connectivity and geometry of chains of tetrahedra, issues (i)–(v) (above) are controlled by other aspects of the structure and details of the environment in which the mineral forms. Here we focus exclusively on the effects of the connectivity and geometry of chains of tetrahedra, and future work will focus on other aspects of structure and the details of the environment of formation.

## 2. Terminology

Following Day & Hawthorne (2020 ▸, 2022 ▸), we define the following terms:

*Chain:* an arrangement of (*T*O_4_)^*n*−^ tetrahedra that (i) links together infinitely in a single direction, (ii) has periodic (translational) symmetry, and (iii) can be broken into two parts by eliminating a single linkage between adjacent tetrahedra.

*Ribbon:* an arrangement of (*T*O_4_)^*n*−^ tetrahedra that (i) links together infinitely in a single direction, (ii) has periodic (translational) symmetry, and (iii) cannot be broken into two parts by eliminating a single linkage between adjacent tetrahedra.

*Tube:* an arrangement of (*T*O_4_)^*n*−^ tetrahedra that (i) links together infinitely in a single direction, (ii) also links orthogonal to the direction of polymerization to form a hollow cylinder, (iii) has periodic (translational) symmetry and (iv) cannot be broken into two parts by eliminating a single linkage between adjacent tetrahedra.

*Cluster:* a 0-dimensional structural unit of linked (*T*O_4_)^*n*−^ tetrahedra that do not extend infinitely in any direction. The graph of a cluster may be *planar* or *non-planar*.

*Structural unit:* the strongly bonded part of a structure, con­sisting of oxyanions and low-coordination-number cations (Hawthorne, 1983*a* ▸, 2015 ▸).

*Backbone chain*: the part of a chain of tetrahedra in which all tetrahedra are connected to form infinite paths (see the definition of path below) in the direction of infinite polymerization.

*Repeat unit:* that part of a chain, ribbon or tube of (*T*O_4_)^*n*−^ tetrahedra that can be repeated by translational symmetry to produce the complete chain, ribbon or tube. To specify the number of 1-, 2-, 3- and 4-connected tetrahedra that comprise the repeat unit of a chain, we denote a tetrahedron by *T*, its connectivity by the superscript *c* (*c* = 1–4) and the number of tetrahedra with connectivity *c* by the subscript *r*. The expression *^c^T_r_* = ^1^ *T_r_* ^2^ *T_r_* ^3^ *T_r_* ^4^ *T_r_* represents all possible connectivities of tetrahedra in the repeat unit of a chain, ribbon or tube.

We refer to ‘chains, ribbons and tubes’ of (*T*O_4_)^*n*−^ tetrahedra as ‘chains’ except where it is necessary to distinguish between chains, ribbons and tubes as defined above. Where we refer to a silicate chain, it must contain Si^4+^ but may also contain other tetrahedrally coordinated cations: *e.g.* *T* = P^5+^, V^5+^, As^5+^, Si^4+^, Al^3+^, Fe^3+^ and B^3+^.

As described in the *Introduction*, Day & Hawthorne (2022 ▸) generated all possible 1-periodic non-isomorphic graphs that contain up to 6–8 symmetrically independent vertices in the topological unit cell. Here, we describe such graphs using the axioms of graph theory as follows:

*Graph:* a graph, *G* = (*V*, *E*), consists of a set of vertices (*V*) and a set of unordered pairs of vertices called edges (*E*).

*Chain graph:* a 1-periodic graphical representation of a chain of (*T*O_4_)^*n*−^ tetrahedra in which tetrahedra are represented as vertices and linkages between tetrahedra are represented as edges. A chain graph contains only the topological information of the corresponding chain of (*T*O_4_)^*n*−^ tetrahedra and does not contain any geometrical information.

*Topological repeat unit:* a subgraph of a chain graph that contains the minimum number (*n*_t_) of non-isomorphic vertices such that it can be repeated by translation along the infinite path of the chain graph to reproduce the chain graph. Analogous to the *^c^T_r_* expression (described above), we specify the connectivity and number of vertices in the topological repeat unit using the *^c^V_r_* expression, *i.e. ^c^V_r_* = ^1^ *V_r_* ^2^ *V_r_* ^3^ *V_r_* ^4^ *V_r_* represents all possible connectivities of vertices in the topological repeat unit of a chain graph. As any unit-distance graph (see definition below) or chain of tetrahedra can be represented topologically as a chain graph, one can also describe the analogous repeat unit of a unit-distance graph or chain of tetrahedra.

*Translational symmetry of graphs:* a 1-periodic chain graph consists of a series of (identical) topological repeat units that extend along an infinite 1-periodic path of the graph. We refer to the topological repeat units as being *translationally equivalent* not because of identical geometric characteristics (as they do not have any geometrical characteristics) but because the topological repeat units along the chain repeat by translation from one repeat unit to another along the infinite path of the chain.

*Cycle (polygon):* a series of vertices and edges that may be traversed such that no vertices or edges are crossed more than once and the traverse ends at the same vertex on which it began. For easy visual recognition, we will refer to a cycle as a polygon in which the dimension of the polygon corresponds to the number of edges (and vertices) in the cycle.

*Cyclic* (polygon) *graph:* a graph that contains one or more cycles (polygons).

*Acyclic* (non-polygon) *graph:* a graph that does not contain cycles (polygons).

*Walk*: a finite or infinite sequence of edges that join a sequence of vertices.

*Path*: a walk in which all vertices (and hence all edges) are distinct.

*Backbone*: the subgraph of a chain graph in which all vertices of that subgraph are linked to form infinite paths in the direction of infinite polymerization.

*Branch*: vertices with linkages that are not part of the backbone. Unlike the backbone, branches contain vertices linked by edges that do not exit the topological repeat unit of the chain graph. *Linear branches* contain vertices that form a finite path and *polygonal branches* contain vertices that form a cycle.

We refer to 1-periodic graphs representative of (isomorphic with) ‘chains, ribbons and tubes’ as *chain graphs* except where it is necessary to distinguish between chains, ribbons and tubes.

As described in the *Introduction*, the chain graphs generated by Day & Hawthorne (2022 ▸) are embedded into 2D and 3D Euclidean space to examine the compatibility of the resultant geometric graphs (*i.e.* unit-distance graphs) with the metrics of crystal structures. Geometric graphs contain spatial information (*e.g.* edge lengths) and thus are described using the axioms of geometric graph theory (Pach, 2004 ▸) as follows:

*Geometric graph:* in the most general sense, a geometric graph is a graph that is defined at least partly by geometric means. A common definition describes a geometric graph as a graph with straight edges occurring in the Euclidean plane. However, for our purposes, we will define a geometric graph as *a graph with straight edges occurring in Euclidean space*.

*Unit-distance graph:* a geometric graph with all edges of unit length; here, we will generalize this definition slightly: all edges will be of equal length. Once a chain graph (or other graph) has been embedded in Euclidean space, it is transformed into a geometric graph; if any graph is embedded with the constraint of equal edges it is a unit-distance graph. It follows that a geometric graph or a unit-distance graph is an embedding of a graph or chain graph.

Strictly speaking, a unit-distance graph is a ‘unit-distance embedding’. However, the expression ‘geometric graph theory’ is now well established in the literature [*e.g.* see the volume by Pach (2004 ▸)] and the terms ‘unit-distance graph’, ‘rigid graph’ *etc*. are common terms in geometric graph theory. We prefer to use the term unit-distance graph rather than unit-distance embedding as it is more intuitive to the crystallographic community.

*Geometrical repeat unit:* that part of a unit-distance graph (or chain of tetrahedra) with the minimum number (*n*_g_) of vertices (or tetrahedra) such that it can be repeated by translational symmetry to produce the complete geometric graph (or chain of tetrahedra). The connectivity of vertices (or tetrahedra) in the geometric repeat unit can be described using the *^c^T_r_* expression (see the definition above).

*Rigid geometric graph:* a geometric graph that cannot produce a different geometric graph by changing the relative positions of its vertices without changing the relative lengths of its edges.

*Flexible geometric graph:* a geometric graph that can produce a different geometric graph by changing the relative positions of its vertices without changing the relative lengths of its edges.

Most geometric graphs dealt with here are unit-distance graphs (as defined above) and when describing the rigidity (or flexibility) of such unit-distance graphs, we refer to them as *rigid unit-distance graphs* and *flexible unit-distance graphs*. Of course, unit-distance graphs may have varying degrees of rigidity (or flexibility) and a method by which this can be quantified is described in Section 6.

## 3. Chain graphs and chains of (*T*O_4_)^*n*−^ tetrahedra in minerals

Most topologically distinct chain graphs generated by Day & Hawthorne (2022 ▸) do not correspond to chains of tetrahedra that occur in minerals (or synthetic compounds), although some proportion of the as-yet unrealized chain graphs may correspond to chains of (*T*O_4_)^*n*−^ tetrahedra that occur in minerals that have not yet been discovered (∼150 new minerals are discovered each year). Day & Hawthorne (2020 ▸) showed that there are ∼50 topologically non-isomorphic chains of tetrahedra in the ∼450 currently approved chain-silicate minerals, four of which occur in ∼375 chain silicates and ∼46 of which occur in ∼75 chain silicates. Furthermore, they showed that there are particular chain stoichiometries (represented by O:*T*, the ratio of oxygen to tetrahedrally coordinated cations) that correspond to minerals (O:*T* = 3.0–2.5), and chain stoichiometries that are not observed in minerals (O:*T* = 2.5–2.0) but correspond to topologically possible chain graphs: *e.g.* O:*T* = 2.0 (^4^ *V*_3–6_ tubes), O:*T* = 2.2 (a ^3^ *V*_2_^4^ *V*_3_ ribbon) and O:*T* = 2.4 (a ^3^ *V*_4_^4^ *V*_1_ ribbon) are possible, although not yet realized in crystal structures (Day & Hawthorne, 2020 ▸).

Although many chains contain *T* cations other than Si^4+^ (*e.g.* Al^3+^, B^3+^, Be^2+^ *etc*.), they all behave similarly with regard to connectivity: all (*T*O_4_)^*n*−^ tetrahedra are 1- to 4-connected and share only corners with other tetrahedra except for a very small number of [Be_2_O_6_] dimers in which tetrahedra share an edge (Hawthorne & Huminicki, 2002 ▸).

## 4. Strategy for embedding chain graphs into Euclidean space

As noted in the *Introduction*, our principal intent is to examine the factors that affect the embedding of chain graphs into 2D and 3D Euclidean space such that the geometrical characteristics of the resultant embedding (unit-distance graph) are compatible with the metrics of crystal structures, metrics involving realistic *T*–O bond lengths and *T*–O–*T* bond angles. Gagné & Hawthorne (2016 ▸, 2018*a* ▸,*b* ▸, 2020 ▸) list ranges of *T*–O and 〈*T*–O〉 bond lengths for the ions of interest here in ∼10 000 well refined, well ordered crystal structures. For mean bond lengths, the dispersion in values is fairly restricted: *e.g*. 1.590–1.658 Å for 〈^[4]^Si^4+^–O^2−^〉 distances with a grand mean value of 1.625 Å. All other cations have mean *T*–O bond lengths within ∼17% of the grand 〈^[4]^Si^4+^–O^2−^〉 value, and the silicate structures *sensu lato* will show a much more restricted range as silicates do not consist entirely of, for example, (B^3+^O_4_)^5−^ (〈^[4]^B^3+^–O^2−^〉 = 1.475 Å) or (Mg^2+^O_4_)^6−^ (〈^[4]^Mg^2+^–O^2−^〉 = 1.939 Å) tetrahedra.

However, the 1-periodic infinite graphs derived by Day & Hawthorne (2022 ▸) do not involve *T*–O linkages (as to do this would drive the derivation beyond computational feasibility), they involve *T*–*T* linkages. However, in crystal structures, *T*–*T* distances involved in *T*–O–*T* linkages are strongly restrained by the *T*–O distances and *T*–O–*T* angles, and hence *T*–*T* distances must have a fairly restricted variation in crystal structures. It is important to note that we are not embedding atoms (entities with metric character, *i.e.* an atomic radius) in Euclidean space but rather chain graphs which contain vertices (entities with no metric character, *i.e.* an infinitely small point) that represent atoms and edges (lines with a set length and no thickness). Therefore, embeddings (unit-distance graphs) can be 1D, 2D or 3D whereas all embeddings of atoms are 3D. We embed chain graphs in only 2D and 3D as only one chain graph (of vertex connectivity ^2^ *V*_1_) can be embedded in 1D without violating *T*–*T* and/or *T*⋯*T* constraints.

### 4.1. *T*–*T* and *T*⋯*T* distances in chain-silicate minerals

When embedding 1-periodic chain graphs into Euclidean space, it is not the exact *T*–*T* distance that is important. For example, if dealing with a borate structure, the *T*–*T* distance should correspond to that in borate structures, whereas dealing with phosphate structures, the *T*–*T* distance should correspond to that in phosphate structures. In each of these types of structures, the *T*–*T* distances are very similar *within* each type of structure, *i.e.* the *T*–*T* distances are approximately the same. Thus, when embedding chain graphs into 2D and 3D Euclidean space, we will use the restraint that *T*–*T* distances must be the same. Of course, real structures can relax from this restraint, but they do not relax very much, *i.e*. the range of *T*–*T* distances will be small in a specific structure and thus the restraint of equal *T*–*T* distances (with a small allowable deviation) seems reasonable.

Equalization of edges (*T*–*T* distances) of a chain graph will also change the distances between pairs of vertices that do not correspond to edges: we will designate such distances as *T*⋯*T* separations. In crystal structures, *T*⋯*T* separations will be significantly longer than *T*–*T* distances as there are no *T*–O bonds restraining the *T*–*T* distance, and such *T*⋯*T* separations cannot become too short without the tetrahedra occupying part of the same volume (in extreme cases) or causing instability of the atomic arrangement by having ions of the same charge too close.

#### 4.1.1. Observed *T*–*T* distances

**Figure 1:**
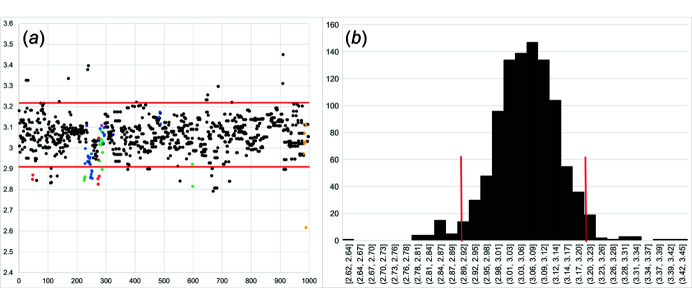
(*a*) A scatter plot and (*b*) a histogram of observed *T*–*T* distances in all chain-silicate minerals and selected synthetic compounds. All symmetrically unique *T*–*T* distances in the geometrical repeat unit of each mineral (or synthetic) were incorporated. Red lines represent the set maximum (3.210 Å) and minimum (2.910 Å) *T*–*T* distances allowed during embedding. In (*a*), black points represent Si^4+^–Si^4+^ distances and the blue, purple, green, red, pink and yellow points represent *T*–*T* distances that involve the *T* cations Al^3+^, V^5+^, Be^2+^, B^3+^, Fe^3+^ and Li^+^, respectively.

Here, we use the data of Day & Hawthorne (2020 ▸) to examine the range of *T*–*T* distances in chain-silicate minerals. The scatter and histogram plots in Figs. 1 ▸(*a*) and 1 ▸(*b*) show the distribution of all *T*–*T* distances in the geometrical repeat (*n*_g_) unit of all chain-silicate minerals and related synthetic compounds. The *T*–*T* distances are in the range 2.616–3.450 Å with an average of 3.060±0.15 Å. Approximately 94% of the *T*–*T* distances are 2.910–3.210 Å (Fig. 1 ▸), and values outside this range tend to involve other tetrahedrally coordinated cations. Thus, when embedding chain graphs, we restrain *T*–*T* distances to 3.060±0.15 Å.

#### 4.1.2. Observed *T*⋯*T* separations

**Figure 2:**
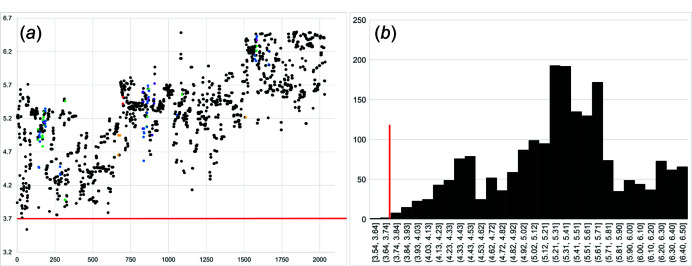
(*a*) A scatter plot and (*b*) a histogram of observed *T*⋯*T* separations in all chain-silicate minerals and selected synthetic compounds. All symmetrically unique *T*⋯*T* separations up to 6.5 Å in the geometrical repeat unit of each mineral (or synthetic) were incorporated. Red lines represent the set minimum (3.713 Å) *T*⋯*T* separations permitted during embedding. In (*a*), black points represent Si⋯Si separations and the blue, purple, green, red, pink and yellow points represent *T*⋯*T* separations that involve the *T* cations Al, V, Be, B, Fe and Li, respectively.

**Figure 3:**
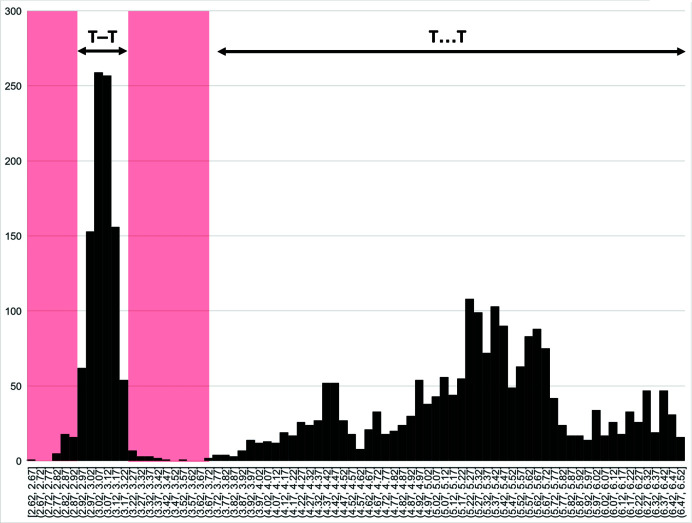
A histogram of observed *T*–*T* distances and *T*⋯*T* separations in all chain-silicate minerals and selected synthetic compounds. The areas in pink from 2.616 to 2.910 Å represent the range in which *T*–*T* distances are not allowed during embedding. The area in pink from 3.210 to 3.713 Å is the range in which *T*–*T* distances and *T*⋯*T* separations are not allowed during embedding.

The scatter and histogram plots in Figs. 2 ▸(*a*) and 2 ▸(*b*) show the distribution of all *T*⋯*T* separations (up to 6.5 Å) in the geometrical repeat unit (*n*_g_) of all chain-silicate minerals and related synthetic compounds. As expected, there is no correlation between the type of *T* cation and *T*⋯*T* separation as unlinked vertices are not restrained by *T*–O bonds. The minimum *T*⋯*T* distance is 3.54 Å [Si–Si in marsturite (Kolitsch, 2008 ▸)] but there are no data between 3.540 and 3.713 Å and only ∼20 data points between 3.713 and 3.904 Å. Hence, when embedding chain graphs, we set the minimum *T*⋯*T* separation to 3.713 Å. For a particular embedding, the minimum difference allowed between *T*–*T* and *T*⋯*T* is 3.713 − 3.210 = 0.503 Å (Fig. 3 ▸, shown in pink). Any chain graph that requires *T*–*T* distances smaller or larger than 3.060±0.15 Å and/or *T*⋯*T* separations smaller than 3.713 Å, once embedded in Euclidean space, is unlikely to occur in minerals. A software program has been developed to apply *T*–*T* and *T*⋯*T* restraints to the chain graphs generated by Day & Hawthorne (2022 ▸); this is discussed in detail below.

### 4.2. Applying the restraint of equal *T*–*T* distances

How do we proceed with applying the restraint of equal *T*–*T* distances when considering the embedding of chain graphs? There is no established way in which this may be done, although there is some software available which can do this for finite graphs in 2D (Rostami *et al.*, 2014*a* ▸,*b* ▸). We wish to understand the properties of graphs that allow or prevent such edge equalization, and hence we will use a combination of visual examination and software minimization of the differences between edge lengths in an embedded graph. Initially, we will proceed by examining a cross section of the chain graphs derived by Day & Hawthorne (2022 ▸).

#### 4.2.1. Acyclic graphs

Let us first consider acyclic chain graphs which consist of a single backbone that may or may not be decorated with *n*-membered linear branches (Day & Hawthorne, 2022 ▸). Acyclic chain graphs are strictly chains as opposed to ribbons and tubes as they may be broken by eliminating a single linkage between adjacent tetrahedra (Day & Hawthorne, 2020 ▸).

**Figure 4:**
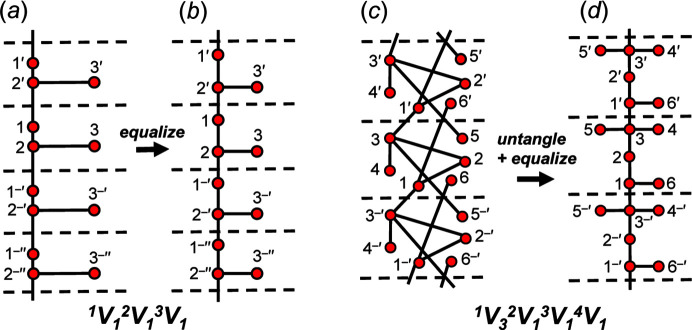
(*a*) An acyclic chain graph; (*b*) the corresponding unit-distance graph now equalized (*i.e*. with edges of equal length); (*c*) an acyclic chain graph; (*d*) the corresponding unit-distance graph after embedding, untangling and with edges of equal length. Vertices are labelled as described in Section 4.2.1 and dashed black lines show the repeat units of each chain.

We are dealing with graphs that have translational symmetry (*e.g.* Fig. 4 ▸) and thus graphs with edges that link translationally equivalent vertices along the infinite 1-periodic path through the chain graph. We need to be able to distinguish between translationally equivalent vertices when describing these graphs, and hence we need a form of labelling that conveys the information that the vertices are translationally equivalent in the +**c** direction and also occur in different unit cells (topological repeat units). We will label such vertices by their integer label in the original unit cell and indicate their translational character by addition of one or more prime symbols, the number of primes indicating the number of translations that relate the original vertex to its translationally equivalent vertices. Translation in the −**c** direction will be indicated by a negative (−) sign before the prime. This notation is shown in Fig. 4 ▸.

**Figure 5:**
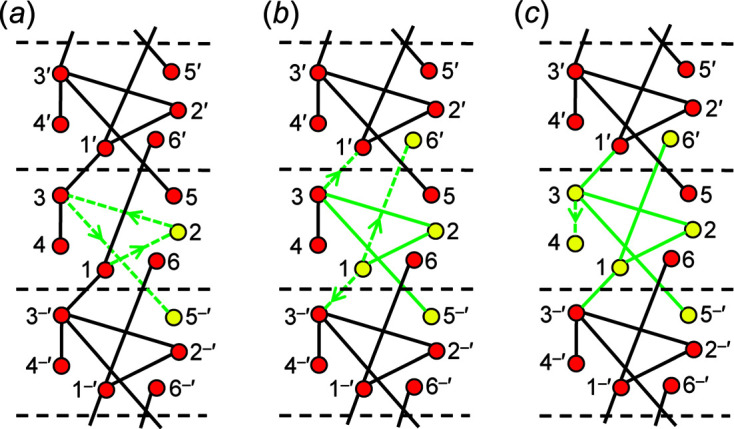
A series of acyclic chain graphs illustrating the heuristic for determining if the edges of an acyclic chain graph can be equalized. Colouring and labelling are explained in Section 4.2.1.

A simple chain graph with vertex connectivity ^1^ *V*_1_^2^ *V*_1_^3^ *V*_1_ is shown in Fig. 4 ▸(*a*) in which there are three different *T*–*T* distances (edge lengths). The *T*–*T* distances may be equalized by changing the relative position of vertices to produce the unit-distance graph in Fig. 4 ▸(*b*). A more complicated chain graph with vertex connectivity ^1^ *V*_3_^2^ *V*_1_^3^ *V*_1_^4^ *V*_1_ is shown in Fig. 4 ▸(*c*); here, it is necessary to untangle the graph first, and then it becomes apparent that the *T*–*T* distances may be equalized as shown in the unit-distance graph in Fig. 4 ▸(*d*). We need a way to test whether an acyclic graph can be equalized. Consider a *walk* in the graph ^1^ *V*_3_^2^ *V*_1_^3^ *V*_1_^4^ *V*_1_ in Fig. 4 ▸(*c*) in which we colour each edge dashed green as it is traversed in the direction of the arrow, set its length to *T*–*T*, and colour each vertex yellow when it has only incident green edges [Fig. 5 ▸(*a*)]. We start on vertex 1 and move to vertex 2, colouring the edge 1–2 dashed green; as vertex 2 is not a component of any other green edges, we may move vertex 2 to set the 1–2 edge length to *T*–*T*, and this action does not affect any other green edges. Next, we move to vertex 3, colouring edge 2–3 dashed green and vertex 2 yellow, and then we move to vertex 5, colouring edge 3–5 green and vertex 5 yellow [Fig. 5 ▸(*a*)]. As there is no red vertex to traverse from vertex 5, we change the dashed green edges to solid green edges and return to the lowest numbered vertex that is still red [vertex 1, Fig. 5 ▸(*a*)]. We traverse edge 1–6, colouring it dashed green and vertex 6 yellow; we return to vertex 1 and traverse edge 1–3′, colouring it dashed green, and colour the translationally symmetric edge 1′′–3 dashed green and vertex 1 yellow. As vertices 1 and 2 are yellow [Fig. 5 ▸(*b*)], we move to vertex 3 and traverse the edge 3–4, colouring it green and the vertices 3 and 4 yellow [Fig. 5 ▸(*c*)]. In this sequence, there is no restraint on setting the lengths of all edges of the graph to *T*–*T*, as all traversed edges that are equalized are initially incident at a red vertex which can be moved without affecting the length of any green (fixed) *T*–*T* edge. This heuristic can apply to any number of vertices in any acyclic graph: hence all acyclic graphs are *equalizable*.

#### 4.2.2. Cyclic graphs

**Figure 6:**
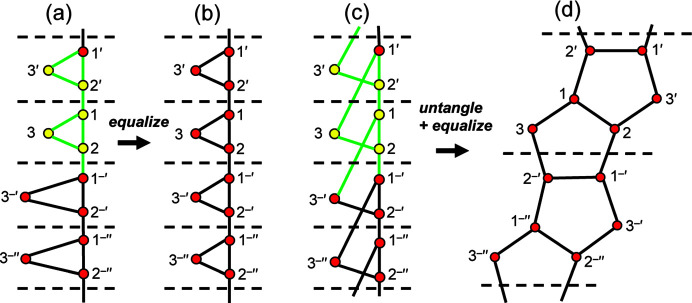
A series of cyclic chain graphs illustrating the equalization and untangling of edges of a cyclic chain graph. Colouring and labelling are explained in Section 4.2.1.

Let us consider the cyclic chain graph with vertex connectivity ^2^ *V*_1_^3^ *V*_2_ in Fig. 6 ▸(*a*) with two distinct *T*–*T* distances [edges 1–2 and 1–3 (or 2–3)]. Using the above heuristic, we begin the walk at vertex 1: 1 → 2′ → 3′ → 1′ → 2′ which results in 1(red), 2′(red), 3′(yellow), 1′(red), 2′(yellow). Starting again at 1′ → 2′′ → 3′′ → 1′′ → 2′′ results in 1′(yellow), 2′′(red), 3′′(yellow), 1′′(red), 2′′(yellow). Thus, all edges of any unit cell can be equalized as shown in Fig. 6 ▸(*a*) and this chain graph is equalizable as shown in Fig. 6 ▸(*b*). Fig. 6 ▸(*c*) shows a more complicated cyclic graph, also with vertex connectivity ^2^ *V*_1_^3^ *V*_2_. Using the above heuristic, we begin the walk at vertex 1: 1 → 2′ → 2′′ → 1′ → 1 which leaves all the vertices red. Starting again at 1′ → 2′′ → 2′′′ → 1′′ → 1′ results in 1′(yellow), 2′′(yellow), 2′′′(red), 1′′(red). Continuing these walks changes all vertices to yellow and all edges to green, and hence the graph is equalizable. Untangling this graph shows the equalized graph to be a chain of edge-sharing pentagons [Fig. 6 ▸(*d*)]. However, note that the number of vertices in the repeat unit of the geometric chain graph in Fig. 6 ▸(*d*) is 6, double the number of vertices (3) in the topological repeat unit of the corresponding chain graph in Fig. 6 ▸(*c*).

**Figure 7:**
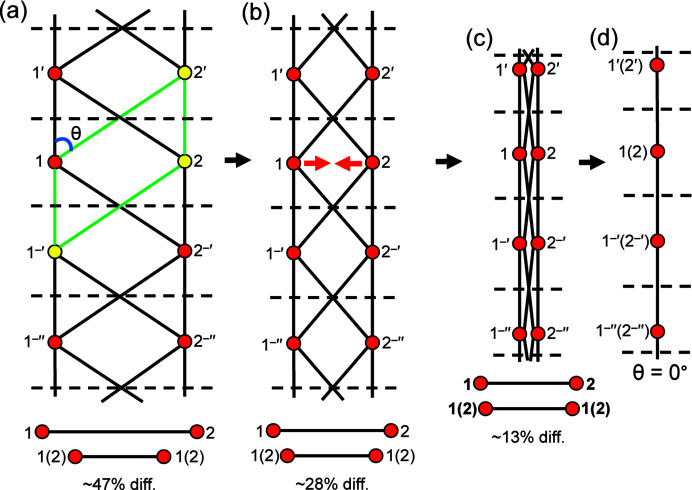
(*a*) A ^4^ *V*_2_ unit-distance graph in which the 1–2 and 1–1 (2–2) edges are of different length; (*b*), (*c*) the unit-distance graph in (*a*) in which vertices 1 and 2 are moved towards each other to reduce the difference in edge lengths; (*d*) the unit-distance graph in (*a*) in which vertices 1 and 2 are moved to the same position to make the edge lengths the same. Note how the 1–2 and 1–1 (2–2) edges cannot be equal until θ = 0° and vertices 1 and 2 occupy the same position, and thus this unit-distance graph is *non-equalizable* in 2D. Colouring and labelling are explained in Section 4.2.1.

The examples given here suggest that all cyclic graphs are equalizable, an important conclusion as it means that we must look elsewhere for the reason why graphs with *T*O_<2.5_ do not form crystal structures (Day & Hawthorne, 2020 ▸). In this regard, it is instructive to examine the cyclic graph ^4^ *V*_2_ [Fig. 7 ▸(*a*)] with two distinct *T*–*T* distances [edges 1–1 (or 2–2) and 1–2]. Using our heuristic approach, we begin the walk at vertex 1: 1 → 1′ → 2′′ → 2′ → 1 which results in 1(red), 1′(red), 2′′(red), 2′(red) and 1(red). Moving to 1′, we begin the next walk: 1′ → 1′′ → 2′′′ → 2′′ → 1′ which results in 1′(yellow), 1′′(red), 2′′′(yellow), 2′′(yellow), 1′(yellow). Thus, the heuristic indicates that the graph is equalizable.

However, if we attempt equalization in a plane, a problem arises. Let us denote the angle 1′–1–2′ as θ, whence 1–1′ = 1 − 2′cos θ. If we progressively decrease θ [Figs. 7 ▸(*b*), 7 ▸(*c*), 7 ▸(*d*)] to reduce the difference in the *T*–*T* distances 1–1 and 1–2, the *T*⋯*T* 1–2 separation [Fig. 7 ▸(*b*)] becomes smaller than the *T*–*T* distances. If 1–1′ = 1–2′ = *T*–*T*, θ = 0° and vertices 1 and 2 must overlap, resulting in a *T*⋯*T* separation of 0 [Fig. 7 ▸(*d*)]. In Section 4.1.2, we allow *T*–*T* distances to differ by ∼5% and *T*⋯*T* separations must be at least ∼16% larger than *T*–*T* distances. We conclude that the chain graph in Fig. 7 ▸(*a*) cannot form a viable infinite chain of (*T*O_4_)^*n*−^ tetrahedra as *T*–*T* distances cannot be made equal (or close to equal) without forcing *T*⋯*T* separations that are 0 (or close to 0). Note that this example is a limiting case; the *T*–*T* distances of most chain graphs may be equalized without reducing *T*⋯*T* separations to 0 (or even close to 0).

### 4.3. Planar and non-planar geometric graphs: the restraint of minimum *T*⋯*T* separations

Any chain graph may be embedded in Euclidean space while applying the restraints of equal *T*–*T* distances and minimum *T*⋯*T* separations to determine if it is compatible with the metrics of chains of *T*O_4_ tetrahedra. Such embeddings result in the following types of unit-distance graphs:

(i) Unit-distance graphs with equal (or almost equal) *T*–*T* distances and *T*⋯*T* separations at least ∼16% larger than all *T*–*T* distances.

(ii) Unit-distance graphs with equal (or almost equal) *T*–*T* distances and *T*⋯*T* separations that are not ∼16% larger than *T*–*T* distances [*e.g.* Figs. 7 ▸(*b*), 7 ▸(*c*), 7 ▸(*d*)].

(iii) Unit-distance graphs with unequal *T*–*T* distances and *T*⋯*T* separations at least ∼16% larger than all *T*–*T* distances [*e.g.* Fig. 6 ▸(*c*)].

Unit-distance graphs of types (ii) and (iii) are unlikely to occur in minerals, whereas unit-distance graphs of type (i) may occur in minerals. Although embedded chain graphs that result in (i) may occur in minerals, they may occur only with specific geometries as the set of all possible geometries that a chain graph may adopt once embedded is restrained by minimum *T*⋯*T* separations.

#### 4.3.1. Acyclic graphs

**Figure 8:**
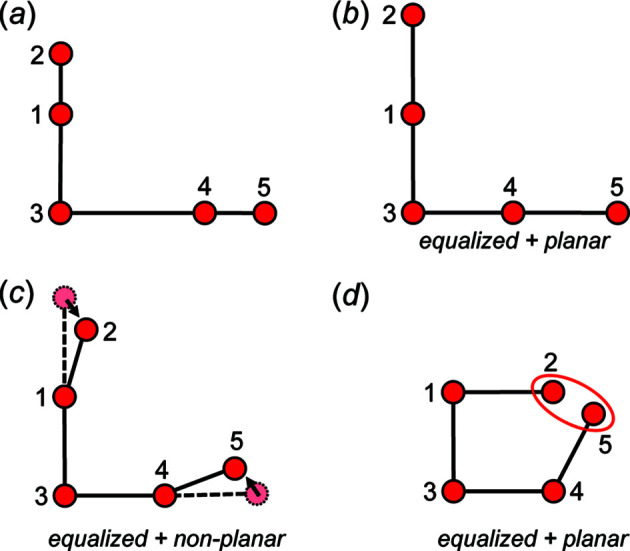
(*a*) A planar finite unit-distance graph with edges of unequal length; (*b*) the unit-distance graph in (*a*) now equalized; (*c*) the unit-distance graph in (*b*) in which vertex 2 is moved out of the plane of the page and vertex 5 is moved into the plane of the page to produce a non-planar unit-distance graph; (*d*) the unit-distance graph in (*b*) in which vertices 2 and 5 are moved such that the *T*⋯*T* separation 2–5 (denoted by the red ellipse) is shorter than the *T*–*T* distance and is not allowed.

Consider the finite acyclic geometric graph in Fig. 8 ▸(*a*). We may equalize the edge lengths to produce the finite unit-distance graph in Fig. 8 ▸(*b*) in which all *T*–*T* distances are equal. The vertices and edges of the unit-distance graph in Fig. 8 ▸(*b*) lie in the plane of the page and no edges cross; thus, it is *planar*. However, we may also produce a *non-planar* equalized unit-distance graph in which vertices 2 and 5 do not lie in the plane of the page and edges 1–2 and 4–5 extend out of the plane of the page [Fig. 8 ▸(*c*)]. There are an infinite number of geometries that this unit-distance graph may adopt, and we conclude that equalization of the edge lengths of this acyclic geometric graph [Fig. 8 ▸(*a*)] does not restrain the geometry of the resultant unit-distance graph. This is the case for all acyclic graphs. One may equalize the geometric graph in Fig. 8 ▸(*a*) to produce the planar geometric graph in Fig. 8 ▸(*d*). However, in this geometric graph, the *T*⋯*T* separation 2–5 is shorter than the *T*–*T* distance and thus this geometry is not physically possible in a chain of tetrahedra embedded in a crystal structure, an example of how minimum *T*⋯*T* separations may restrain the geometry of acyclic geometric graphs.

#### 4.3.2. Cyclic graphs

**Figure 9:**
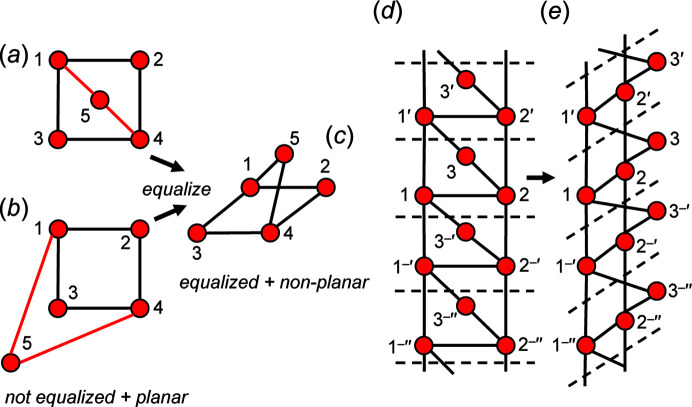
A planar finite unit-distance graph in which the 1–5 and 4–5 edges (shown in red) are (*a*) shorter and (*b*) longer than the other edges; (*c*) the unit-distance graph in (*a*) and (*b*) now equalized; this unit-distance graph is forced into a non-planar arrangement by equalizing the 1–5 and 4–5 edges; (*d*) a planar unit-distance graph in which the 1–3 and 2–3 edges are shorter than the 1–1, 1–2 and 2–2 edges; (*e*) the unit-distance graph in (*d*) now equalized; it is forced into a non-planar arrangement by equalizing the 1–3 and 2–3 edges. Vertices are labelled as described in Section 4.2.1 and dashed black lines show the repeat units of each chain.

Consider the cyclic geometric graphs in Figs. 9 ▸(*a*) and 9 ▸(*b*). These geometric graphs are planar but have unequal *T*–*T* distances: the red edges are either shorter [Fig. 9 ▸(*a*)] or longer [Fig. 9 ▸(*b*)] than the black edges. There is no way to equalize the *T*–*T* distances in Figs. 9 ▸(*a*) and 9 ▸(*b*) without (i) crossing edges and/or overlapping vertices (which reduces the *T*⋯*T* separation 5–2 or 5–3 to zero), or (ii) moving vertices out of the plane of the page. Thus, equalizing the *T*–*T* distances of both geometric graphs must result in a non-planar unit-distance graph [Fig. 9 ▸(*c*)]. We conclude that equalizing the edge lengths of the geometric graphs in Figs. 9 ▸(*a*) and 9 ▸(*b*) while maintaining planarity is not possible [Fig. 9 ▸(*c*)] and we refer to such geometric graphs as *planar non-equalizable*. These geometric graphs may be linked to form 1-periodic unit-distance graphs [Fig. 9 ▸(*d*)] that are also forced into a non-planar arrangement when the *T*–*T* distances are equalized [Fig. 9 ▸(*e*)]. Throughout this paper, planar equalized unit-distance graphs are drawn with edges of equal length; non-planar equalized unit-distance graphs may not appear to have equal edges due to depth perspective.

It is important to differentiate *topological planarity* and *geometrical planarity*. Topological planarity is defined by Fáry’s theorem (Fáry, 1948 ▸) which states that any planar graph can be drawn without edge crossings so that its edges are straight-line segments (Chartrand *et al.*, 2010 ▸). Note that there is no requirement for vertices to lie in a Euclidean plane as this theorem applies to graphs which have no geometrical properties. According to Fáry’s theorem, all chain and ribbon graphs are planar, and all tube graphs are non-planar as they cannot be drawn without at least two edges crossing (Day & Hawthorne, 2020 ▸). Geometric planarity describes the planarity of geometric graphs (and unit-distance graphs), which have geometric properties (*e.g.* specified *T*–*T* distances). Here, the relative position of vertices is of concern and the vertices of geometrically planar graphs must lie in the same Euclidean plane, otherwise they are geometrically non-planar (Barthélemy, 2011 ▸). For example, according to Fáry’s theorem, the graph in Fig. 9 ▸(*e*) is topologically planar as it can be drawn without edge crossings [*e.g.* Fig. 9 ▸(*d*)], but is geometrically non-planar as all vertices do not lie in the same Euclidean plane. When we use the terms *planar* and *non-planar* (as done above), we are referring to geometric planarity unless otherwise stated.

## 5. Drawing chain graphs: colouring and labelling of vertices and edges

Chain graphs with a topological repeat unit (*n*_t_) that contains more than 6 vertices are often visually complex and difficult to comprehend. It is important that they be drawn in a way that allows accurate identification of different aspects of the chain (*e.g.* backbones, branches, polygons). To this end, we develop a colour scheme for both vertices and edges that allows easy visual identification of the different constituents of chain graphs irrespective of their degree of entanglement.

**Figure 10:**
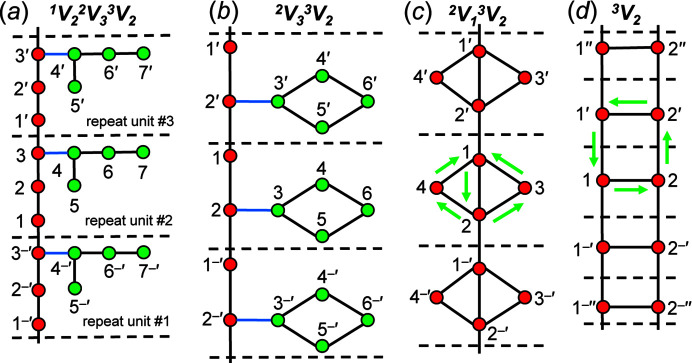
A series of examples showing the colouring and labelling schemes used for drawing chain graphs and the method by which polygons are identified. (*a*) An acyclic chain graph in which vertices that comprise the backbone chain and linear branches are shown in red and green, respectively, edges linking the backbone chain to a branch are shown in blue. (*b*) A cyclic chain graph with polygonal branches drawn as described in (*a*). (*c*) A cyclic chain graph in which the cycles 1–2–3–1 and 1–2–4–1 (shown with green arrows) define triangles and the cycle 1–3–2–4–1 defines a square. (*d*) A cyclic chain graph in which the cycle 1–2–2′–1′–1 defines a square. In (*d*), note that the vertex-labelling scheme allows identification of polygons that span multiple repeat units. Vertices are labelled as described in Section 4.2.1 and dashed black lines show the repeat units of each chain graph.

Consider the acyclic chain graph in Fig. 10 ▸(*a*) with vertex connectivity ^1^ *V*_2_^2^ *V*_3_^3^ *V*_2_. The path 1^–^′ → 2^–^′ → 3^–^′ → 1 → 2 → 3 → 1′ → 2′ → 3′…*etc*. is an infinite path in the direction of chain polymerization and thus vertices 1–3 and the edges linking such vertices comprise the backbone and are coloured red and black, respectively. The path 5 → 4 → 6 → 7 is a finite path (not a cycle) which does not include any edges that exit the repeat unit and thus vertices 4–7 and the edges linking those vertices comprise a linear branch and are coloured green and black, respectively. Notice that the 3–4 edge is not included in either path as this edge links the linear branch to the backbone and is coloured blue. In Fig. 10 ▸(*b*), the path 1^–^′ → 2^–^′ → 1 → 2 → 1′ → 2′…*etc*. defines the backbone and the cycle 3 → 4 → 6 → 5 does not include any edges that exit the topological repeat unit and thus vertices 3–6 and the edges linking those vertices comprise a 4-membered polygonal branch and are coloured green and black, respectively.

The vertices of chain graphs may be labelled as described in Section 4.2.1. This labelling scheme allows identification of infinite paths (backbones) as above and/or polygons (cycles) that span more than one topological repeat unit. Consider the cyclic chain graph in Fig. 10 ▸(*c*) with vertex connectivity ^2^ *V*_1_^3^ *V*_2_. This graph contains triangles defined by the cycles 1–2–3–1 and 1–2–4–1 (shown with green arrows) and a square defined by the cycle 1–3–2–4–1. These polygons are cycles as no vertex or edge is crossed more than once. In Fig. 10 ▸(*d*), a cyclic chain graph with vertex connectivity ^3^ *V*_2_ is shown and a square is defined by the cycle 1–2–2′–1′–1. However, this cycle involves crossing translationally equivalent vertices (2) and edges (1–2) more than once, but such vertices and edges belong to different (adjacent) repeat units and are different (vertices labelled 2 and 2′ and edges labelled 1–2 and 1′–2′). Thus 1–2–2′–1′–1 is a valid cycle, reinforcing the need for such a labelling scheme.

## 6. Rigid and flexible geometric graphs

In Figs. 7 ▸, 8 ▸ and 9 ▸, we show how the geometry (planarity) of some unit-distance graphs is affected by equalizing the edge lengths of their parent cyclic chain graphs. Following the axioms of *topological restraint theory*, commonly known as *rigidity theory* (Asimow & Roth, 1978 ▸; Crapo, 1979 ▸; Thorpe, 1983 ▸; Sen & Mason, 2019 ▸), we may show how the geometry (in both 2D and 3D) of cyclic unit-distance graphs is restrained by a property called *rigidity*, or conversely, *flexibility*. Here we are concerned only with geometric restraints due to equal *T*–*T* distances; geometric restraints due to *T*⋯*T* separations will be discussed later.

**Figure 11:**
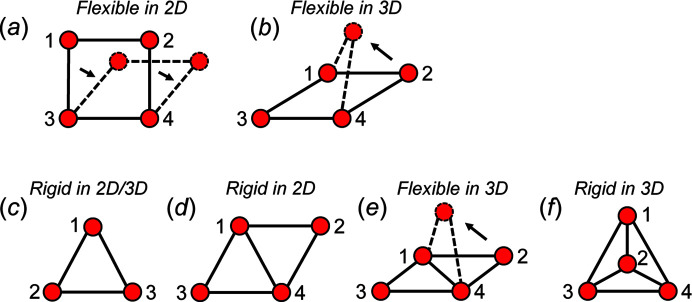
(*a*) A planar square unit-distance graph that is flexible in 2D as vertices 1 and 2 can be moved in the plane of the square to produce a unit-distance graph that is geometrically distinct from the original unit-distance graph (a rhombus rather than a square) while retaining equal edge lengths. (*b*) The square unit-distance graph in (*a*) viewed in the plane of the page. This unit-distance graph is also flexible in 3D as vertex 2 can be moved out of the plane of the square to produce a geometrically distinct unit-distance graph while retaining equal edge lengths. (*c*) A triangular unit-distance graph that is rigid in 2D and 3D as no vertex (or combination of vertices) can be moved to produce a geometrically distinct unit-distance graph without forcing edges to be of unequal length. (*d*) A unit-distance graph that is rigid in 2D but (*e*) flexible in 3D. (*f*) A tetrahedron unit-distance graph that is rigid in 2D and 3D. Dashed black lines and arrows show the movement of vertices 1 and 2 in (*a*) and vertex 2 in (*b*) and (*e*).

Consider the square unit-distance graph in Fig. 11 ▸(*a*). Vertices 1 and 2 may be moved within the plane of the page to form a rhombus (indicated by the dashed lines) while retaining the length of the 1–2, 1–3 and 2–4 edges, and thus this graph is flexible in 2D. In Fig. 11 ▸(*b*), the same unit-distance graph is shown where vertex 2 is moved into the third dimension while retaining the 1–2 and 2–4 edge lengths; thus this unit-distance graph is flexible in 3D. Consider the unit-distance graph in Fig. 11 ▸(*c*); there is no way to move any of the vertices to produce a geometrically distinct triangle (*i.e.* a non-equilateral triangle) without making at least one of the edges shorter (or longer) than the others; thus, this unit-distance graph is rigid. Minimally rigid unit-distance graphs in 2D are commonly referred to as *Laman* graphs and must have 2*n* − 3 edges, where *n* is the number of vertices (Laman, 1970 ▸). One may convert any minimally rigid unit-distance graph into a flexible graph by removing one edge. In rigidity theory, it is not required that all rigid geometric graphs have equal edges, only that the lengths of their edges do not change, and thus all edges are equalized before testing for rigidity. The triangle unit-distance graph [Fig. 11 ▸(*c*)] is one of the simplest examples of a Laman graph and may be combined with other triangles or squares to produce the other Laman graphs. For example, if two equilateral triangles share an edge [Fig. 11 ▸(*d*)], the unit-distance graph is rigid within the plane of the page (Laman, 1970 ▸; Haas *et al.*, 2005 ▸).

If one replaces each vertex with a hinge and edges with rigid rods of equal length, it is not possible to move the hinge in the plane of the page (in 2D) without moving the entire unit-distance graph. One may move a hinge into or out of the plane of the page (*i.e.* in 3D) but this would not result in a geometrically distinct unit-distance graph. The unit-distance graph in Fig. 11 ▸(*d*) consists of two edge-sharing triangles and is rigid in 2D but not in 3D as vertex 2 may be moved into the third dimension while retaining equal edge lengths [Fig. 11 ▸(*e*)]. Rigid geometric graphs in 3D are an open problem in distance geometry and geometric graph theory, but it has been shown that most (but not all) rigid geometric graphs have 3*n* − 6 edges (Grasegger *et al.*, 2018 ▸) in 3D. If an edge is added to the unit-distance graph in Fig. 11 ▸(*d*) that links vertices 2 and 3, a tetrahedron unit-distance graph is produced [Fig. 11 ▸(*f*)] which is rigid in 2D and 3D.

### 6.1. Modes of geometric modification

If any number of vertices of a geometric graph *G* are moved to produce a geometric graph *G*′ that is geometrically distinct from *G*, then *G* has been geometrically modified. The set of vertices moved to produce *G*′ is a *mode of geometric modification* or, more simply, a *mode*, and different modes are generated by moving different combinations of vertices. For most geometric graphs, an infinite number of geometrically distinct (but topologically identical) graphs may be generated by a finite number of modes. Consider a geometric graph with *n* vertices, all of which are equivalent. Moving one vertex is equivalent to moving any other vertex, and hence there is only one mode that involves moving one vertex. Similarly, all pairs of vertices (doublets) are equivalent and there is only one mode involving moving a pair (doublet) of vertices, and similarly for any tuplet of vertices. For *n* vertices, there are *n* tuplets and hence *n* possible modes for a geometric graph with *n* equivalent vertices. Consider a geometric graph with *n* vertices, none of which are equivalent. Moving one vertex is distinct from moving any other vertex, moving any doublet of vertices is distinct from moving any other doublet of vertices, and similarly for any tuplet of vertices. In this case, the number of distinct modes is given by the partition of *n*: *p*(*n*). Thus, symmetry relations among the vertices decrease the number of modes according to the details of the symmetry operations involved.

All possible modes for a given finite geometric graph may be generated using the vertex subsets (see Day & Hawthorne, 2022 ▸) of the underlying graph and are considered valid if they produce geometrically distinct geometric graphs (with respect to the original geometric graph) without changing the length of any edge (*i.e*. equal edge lengths are retained for unit-distance graphs). A mode is considered invalid if it requires that edge lengths be unequal or if it does not produce a geometrically distinct geometric graph (with respect to the original geometric graph).

### 6.2. 2D and 3D modes

Modes may be 2D or 3D and involve moving vertices in the plane or out of the plane of the geometric graph (or the page), respectively. However, some geometric graphs are non-planar and do not define a Euclidean plane; such geometric graphs have only 3D modes. A 3D mode of a non-planar geometric graph will always produce a non-planar geometric graph. It follows that only planar geometric graphs have 2D modes, and that a valid 2D mode will always produce a planar geometric graph. However, a 3D mode of a planar geometric graph may produce a planar or non-planar geometric graph. An invalid 2D or 3D mode of a planar geometric graph may produce a graph that is geometrically identical to the original geometric graph but is rotated in 2D or 3D space with respect to the original geometric graph. Such modes are referred to as *rotational modes*. There are three possibilities for rotational modes:

(i) A valid 2D mode that produces a geometric graph that is geometrically identical to the original but rotated in the plane of the original geometric graph.

(ii) A valid 3D mode of a planar geometric graph that produces a geometric graph that is geometrically identical to the original but rotated such that vertices lie on a different plane than those of the original geometric graph.

(iii) A valid 3D mode of a non-planar geometric graph that produces a geometric graph that is geometrically identical to the original but rotated into an orientation different from that of the original geometric graph.

It follows that there are two types of invalid modes: (i) those that force unequal edge lengths, and (ii) those that are rotational modes.

**Figure 12:**
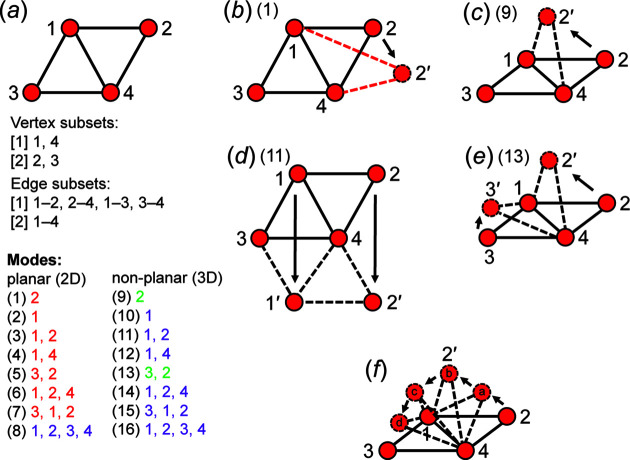
(*a*) A planar unit-distance graph, the corresponding vertex and edge subsets, and the 2D and 3D modes of geometric modification. Mode (1) involves moving vertex 2 in 2D (in the plane of the unit-distance graph) as shown in (*b*) and requires that the 1–2 and 2–4 edges are of unequal length (red dashed lines), and thus mode (1) is *invalid*. Mode (9) involves moving vertex 2 in 3D (out of the plane of the unit-distance graph) as shown in (*c*) and results in a geometrically distinct unit-distance graph while retaining equal edge lengths, and thus mode (9) is *valid*. Mode (11) involves moving vertices 1 and 2 in 3D as shown in (*d*). Here, equal edge lengths are retained but a geometrically distinct unit-distance graph is not produced; instead, the unit-distance graph is rotated in 3D, and thus mode (11) is a *rotational* mode. Mode (13) involves moving vertices 2 and 3 in 3D as shown in (*e*), and results in a geometrically distinct unit-distance graph while retaining equal edge lengths, and thus mode (13) is *valid*. (*f*) Four geometrically distinct graphs that correspond to mode (9) and the movement of vertex 2 to positions ‘a’, ‘b’, ‘c’ and ‘d’. Each valid mode corresponds to an infinite number of geometrically distinct unit-distance graphs. Dashed black lines and arrows show the movement of a vertex labelled *n* to the position *n*′. Valid, invalid and rotational modes are shown in green, red and purple, respectively.

Consider the geometric graph in Fig. 12 ▸(*a*); here all edges are of equal length and we can specify this geometric graph as a unit-distance graph. The vertex subsets are used to derive all possible 2D and 3D modes: vertices 1 and 4 are isomorphic and vertices 2 and 3 are isomorphic, reducing the number of different modes. For example, mode (9) involves vertex 2; there is no mode listed that involves vertex 3 as vertices 2 and 3 are isomorphic. Similarly, mode (15) involves moving vertices 3, 1 and 2 and there is no mode listed that involves moving vertices 3, 4 and 2, as vertices 1 and 4 are isomorphic. In Fig. 12 ▸(*b*), mode (1) is applied and any movement of vertex 2 (or 3) in 2D results in edges of unequal length (red dashed edges) and thus this mode is invalid (and shown in red). In Fig. 12 ▸(*c*), mode (9) is applied and vertex 2 is moved out of the plane of the page to produce a geometrically distinct unit-distance graph while retaining equal edge lengths and thus this mode is valid and shown in green. In Fig. 12 ▸(*d*), mode (11) is applied and vertices 1 and 2 are moved out of the plane of the page (in 3D) to produce a unit-distance graph that is geometrically identical to the original graph but is rotated 180°; thus (11) is a rotational mode (and is shown in purple). Although the unit-distance graph produced by mode (11) is planar [Fig. 12 ▸(*d*)], it cannot be produced by the invalid mode (3) as moving vertices 1 and 2 to the positions of vertices 1′ and 2′ in the plane of the page (in 2D) would involve shortening edges at intermediate positions. It follows that any valid mode must retain equal edge lengths for any position of the vertices associated with that mode. In Fig. 12 ▸(*e*), mode (13) is applied and vertices 2 and 3 are moved out of the plane of the page to produce a unit-distance graph that is geometrically distinct from the original unit-distance graph while retaining equal edge lengths; thus (13) is a valid mode. Each valid mode corresponds to an infinite number of geometrically distinct unit-distance graphs as vertices may be moved to different positions while retaining equal edge lengths. For example, consider the unit-distance graph in Fig. 12 ▸(*f*); vertex 2 may be moved out of the plane of the page through an infinite number of non-equivalent positions, each with different 2′–4–3 angles, starting at 180° and becoming progressively smaller through positions ‘a’–‘d’ [Fig. 12 ▸(*f*)].

### 6.3. Rigid and flexible unit-distance graphs

By comparing the number of valid and invalid modes for a set of unit-distance graphs, we can gauge the relative degree of rigidity for a unit-distance graph with *m* modes, *m*_v_ valid modes and *m*_i_ invalid modes. We define four rigidity groups: (i) if *m*_i_ = *m*, it is rigid; (ii) if *m*_v_ = *m*, it is flexible; (iii) if *m*_i_ ≥ ½*m*, it is semi-rigid; (iv) if *m*_i_ < ½*m*, it is semi-flexible.

We may further differentiate the degree of rigidity of a set of unit-distance graphs by appending *m*_i_/*m* to the rigidity group; thus in Fig. 12 ▸(*a*), the unit-distance graph is *14/16-semi-rigid* and is more rigid than a *12/16-semi-rigid* unit-distance graph. One may also describe the degree of rigidity with respect to planarity; for example, the unit-distance graph in Fig. 12 ▸(*a*) is *8/8-rigid* in 2D (all 2D modes are invalid) and *6/8-semi-rigid* in 3D. It follows that a *0/6-flexible* unit-distance graph (*m*_v_ = *m* = 6) has a higher degree of flexibility than a *0/4-flexible* unit-distance graph as the first unit-distance graph may be geometrically modified by six unique modes compared with four unique modes for the second unit-distance graph, and the corresponding structure in a mineral will have more geometrical freedom to deform in response to stress (temperature, pressure, chemical substitution *etc*.).

### 6.4. Degree of rigidity and flexibility of unit-distance graphs

This approach can now be applied to infinite chain graphs and the vertex subsets for the corresponding directed proto-graph [see Day & Hawthorne (2022 ▸) for the definition of directed proto-graph] may be used to determine the modes of geometric modification. However, as chain graphs and the unit-distance graphs they correspond to are infinite, we must also consider different types of medium-range modification (*e.g.* modulation) that may occur in chains. With respect to the original unit distance *G*, a geometrical modification that produces a unit-distance *G*′ may be classified as:

(i) A *short-range modification:* for a given mode, a vertex (or vertices) is moved in every repeat unit to symmetrically equivalent positions such that *n*_t_ = *n*_g_, or

(ii) A *medium-range modification*: for a given mode, a vertex (or vertices) is not moved in every repeat unit or not moved to equivalent positions in every repeat unit such that *n*_t_ ≠ *n*_g_, and *n*_g_ is a multiple of *n*_t_ (giving rise to chain kinking, modulation, spiralling *etc*.).

**Figure 13:**
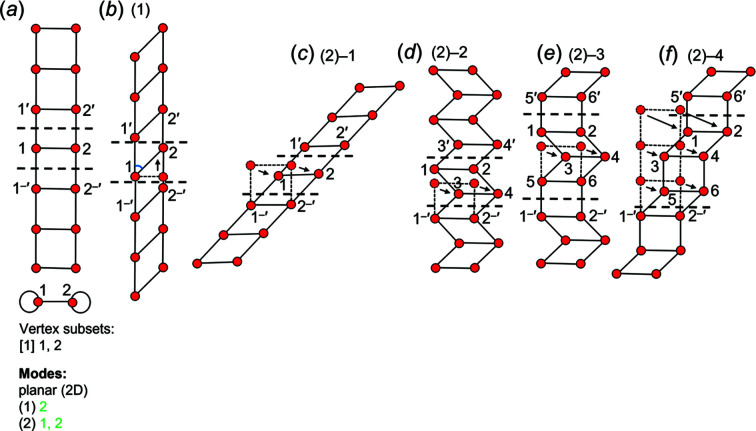
(*a*) A unit-distance graph, the corresponding directed proto-graph, vertex subsets and the 2D modes. Mode (1) involves moving vertex 2 in 2D as shown in (*b*) to produce a planar unit-distance graph that is geometrically distinct from the original unit-distance graph in (*a*). Mode (2) involves moving vertices 1 and 2 in 2D as shown in (*c*) to produce a planar unit-distance graph that is geometrically identical to that shown in (*b*). Mode (2) may be applied to every second or third repeat unit as shown in (*d*) and (*e*), respectively, to produce two planar unit-distance graphs that are both geometrically distinct from the original unit-distance graph in (*a*). Mode (2) may also be applied by moving vertices 1 and 2 to symmetrically non-equivalent positions in each repeat unit as shown in (*f*) to produce an additional geometrically distinct planar unit-distance graph. Both 2D modes result in planar unit-distance graphs that are geometrically distinct from the original unit-distance graph in (*a*) and are therefore valid. Legend as in Figs. 10 ▸ and 12 ▸.

Consider the unit-distance graph of edge-sharing squares and the corresponding directed proto-graph in Fig. 13 ▸(*a*). There are four valid modes: mode (1) involves moving vertex 2 in 2D, converts each square into a rhombus and results in the geometrically distinct unit-distance graph in Fig. 13 ▸(*b*). This unit-distance graph shows short-range modification as vertex 2 is moved to the same symmetrically equivalent position in every topological repeat unit such that *n*_t_ = *n*_g_ = 2. As explained for Fig. 12 ▸(*f*), any valid mode corresponds to an infinite number of unit-distance graphs, and we may move vertex 2 in 2D such that the 1–1–2 (or 1–2–2) angle [Fig. 13 ▸(*b*)] is any value other than 180°, and thus mode (1) corresponds to an infinite number of short-range modifications. Mode (2) involves moving vertices 1 and 2 in 2D and produces the short-range modified unit-distance graph in Fig. 13 ▸(*c*). This unit-distance graph is geometrically identical to the unit-distance graph in Fig. 13 ▸(*b*) but rotated in the plane of the unit-distance graph. However, mode (2) is not a rotational mode as it may be applied to the unit-distance graph in Fig. 13 ▸(*a*) at different intervals (repeat units) to produce additional geometrically distinct unit-distance graphs that show medium-range modification. For example, if mode (2) is applied to every second repeat unit of the unit-distance graph in Fig. 13 ▸(*a*), the geometrically distinct unit-distance graph in Fig. 13 ▸(*d*) is produced with a geometrical repeat unit that contains 4 vertices (*n*_g_ = 4) rather than 2 (*n*_t_ = 2). If mode (2) is applied to every third repeat unit of the unit-distance graph in Fig. 13 ▸(*a*), the geometrically distinct unit-distance graph in Fig. 13 ▸(*e*) is produced with a geometrical repeat unit with *n*_g_ = 6. By applying mode (2) to every fourth, fifth, sixth *etc*. repeat unit, unit-distance graphs with *n*_g_ = 8, 10, 12 *etc*. are produced. Additional geometrically distinct unit-distance graphs are produced by applying mode (2) to different combinations of repeat units and/or by moving vertices 1 and 2 to symmetrically non-equivalent positions in 2D with respect to the other repeat units, *i.e.* forming a chain of edge-sharing squares and rhombuses [Fig. 13 ▸(*f*)]. The unit-distance graphs in Figs. 13 ▸(*d*), 13 ▸(*e*) and 13 ▸(*f*) show medium-range modification.

**Figure 14:**
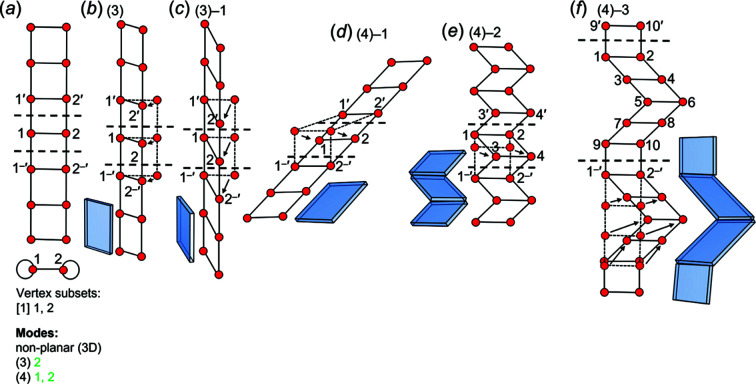
(*a*) A unit-distance graph [isomorphic with the unit-distance graph in Fig. 13 ▸(*a*)], the corresponding directed proto-graph, vertex subsets and the 3D modes. Mode (3) involves moving vertex 2 in 3D and may result in a geometrically identical (*b*) or distinct (*c*) planar unit-distance graph with respect to the original unit-distance graph in (*a*). Mode (4) involves moving vertices 1 and 2 in 3D as shown in (*d*) to produce a planar unit-distance graph that is geometrically identical to that shown in (*a*) and (*b*). Mode (4) may be applied to every second repeat unit as shown in (*e*) to produce a non-planar unit-distance graph that is geometrically distinct from the original unit-distance graph in (*a*). Mode (4) may also be applied by moving vertices 1 and 2 to symmetrically non-equivalent positions in each repeat unit as shown in (*f*) to produce an additional geometrically distinct non-planar unit-distance graph. Both 3D modes result in planar and non-planar unit-distance graphs that are geometrically distinct from the original unit-distance graph in (*a*) and are therefore valid. Blue 3D sheets show the geometry and orientation of each unit-distance graph. Legend as in Figs. 10 ▸ and 12 ▸.

Mode (3) involves moving vertex 2 of the unit-distance graph in Fig. 14 ▸(*a*) in 3D and results in the unit-distance graph in Fig. 14 ▸(*b*) that is geometrically identical to that in Fig. 14 ▸(*a*) and the unit-distance graph in Fig. 14 ▸(*c*) that is geometrically distinct from the unit-distance graph in Fig. 14 ▸(*a*) but identical to unit-distance graphs in Figs. 13 ▸(*b*) and 13 ▸(*c*). As is the case for mode (1), mode (3) must involve moving vertex 2 of every repeat unit to symmetrically equivalent positions; if this is not done, the edge lengths of the unit-distance graph become unequal, and mode (3) does not correspond to any unit-distance graphs that show medium-range modification. Mode (4) involves moving vertices 1 and 2 in 3D; if vertices 1 and 2 of every repeat unit are moved to symmetrically equivalent positions, the resulting unit-distance graph [Fig. 14 ▸(*d*)] is geometrically identical to the unit-distance graph in Fig. 14 ▸(*a*) but is rotated into the plane of the page. However, like mode (2), mode (4) is not a rotational mode as vertices 1 and 2 may be moved to symmetrically non-equivalent positions at different intervals (repeat units) to produce additional geometrically distinct unit-distance graphs that show medium-range modification. For example, if mode (4) is applied to every second repeat unit of the unit-distance graph in Fig. 14 ▸(*a*), the geometrically distinct unit-distance graph in Fig. 14 ▸(*e*) is produced for which *n*_g_ = 4. The geometric unit-distance graph in Fig. 14 ▸(*f*) also shows medium-range modification and is produced by moving vertices 1 and 2 to non-equivalent positions in groups of three adjacent repeat units separated by groups of two repeat units in which vertices 1 and 2 are not moved. We conclude that the unit-distance graph in Fig. 13 ▸(*a*) is *0/4-flexible* as all 2D and 3D modes are valid.

**Figure 15:**
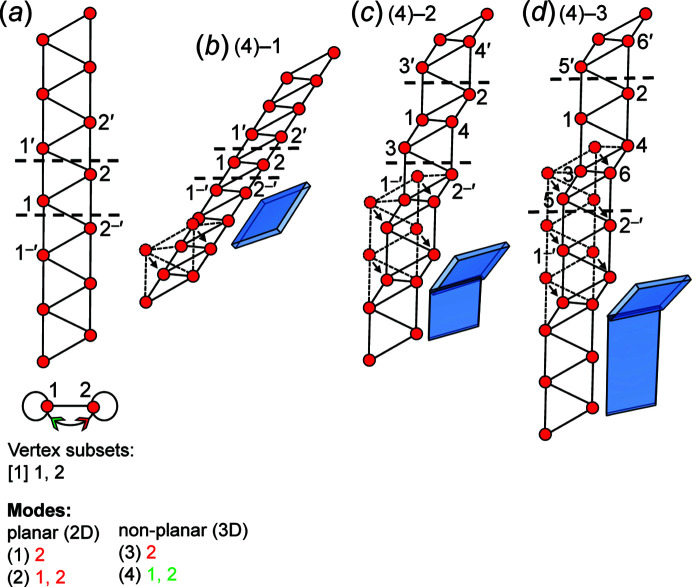
(*a*) A unit-distance graph, the corresponding directed proto-graph, vertex subsets and the 2D and 3D modes. Modes (1), (2) and (3) cannot be applied to produce unit-distance graphs that are geometrically distinct from the original unit-distance graph in (*a*) without forcing unequal edge lengths and are therefore invalid. Mode (4) involves moving vertices 1 and 2 in 3D as shown in (*b*) to produce a planar unit-distance graph that is geometrically identical to the original unit-distance graph in (*a*). Mode (4) may be applied to every second repeat unit as shown in (*c*) to produce a non-planar unit-distance graph that is geometrically distinct from the original unit-distance graph in (*a*). Mode (4) may also be applied by moving vertices 1 and 2 to symmetrically non-equivalent positions in each repeat unit as shown in (*d*) to produce an additional geometrically distinct non-planar unit-distance graph, and therefore mode (4) is valid. Blue 3D sheets show the geometry and orientation of each unit-distance graph. Legend as in Figs. 10 ▸ and 12 ▸.

Consider the unit-distance graph consisting of edge-sharing triangles and its corresponding directed proto-graph in Fig. 15 ▸(*a*). Here, modes (1), (2) and (3) are invalid and mode (4) is valid. Mode (4) involves moving vertices 1 and 2 in 3D; if vertices 1 and 2 of every repeat unit are moved to symmetrically equivalent positions, the unit-distance graph in Fig. 15 ▸(*b*) is produced which is geometrically identical to the unit-distance graph in Fig. 15 ▸(*a*) but is rotated in the plane of the page. If mode (4) is applied to every second repeat unit of the unit-distance graph in Fig. 15 ▸(*a*) and vertices 1 and 2 are moved to symmetrically non-equivalent positions, the geometrically distinct unit-distance graph in Fig. 15 ▸(*c*) is produced in which *n*_g_ = 4. If mode (4) is applied to every third repeat unit of the unit-distance graph in Fig. 15 ▸(*a*) and vertices 1 and 2 are moved to symmetrically non-equivalent positions, the geometrically distinct unit-distance graph in Fig. 15 ▸(*d*) is produced for which *n*_g_ = 6. We conclude that the unit-distance graph in Fig. 15 ▸(*a*) is *3/4-semi-rigid*.

### 6.5. Degree of unit-distance graph rigidity and the *e*/*n* ratio

The mathematical conditions for rigidity of infinite geometric graphs are not known. However, as the ratio of the number of edges to the number of vertices (*e*/*n*) increases, the degree of rigidity of a geometric graph commonly increases (and flexibility decreases). For example, consider the unit-distance graph in Fig. 11 ▸(*a*) which is semi-rigid in 2D and 3D with *e*/*n* = 1.0. If a 1–4 edge is added to this unit-distance graph, *e*/*n* = 1.25 and the unit-distance graph in Fig. 11 ▸(*d*) is produced which is rigid in 2D and semi-rigid in 3D. If a 2–3 edge is added to the unit-distance graph in Fig. 11 ▸(*d*), *e*/*n* = 1.5 and the unit-distance graph in Fig. 11 ▸(*f*) is produced which is rigid in both 2D and 3D. Thus, for the examples in Figs. 11 ▸(*a*), 11 ▸(*d*) and 11 ▸(*f*), the degree of rigidity increases with *e*/*n*.

**Figure 16:**
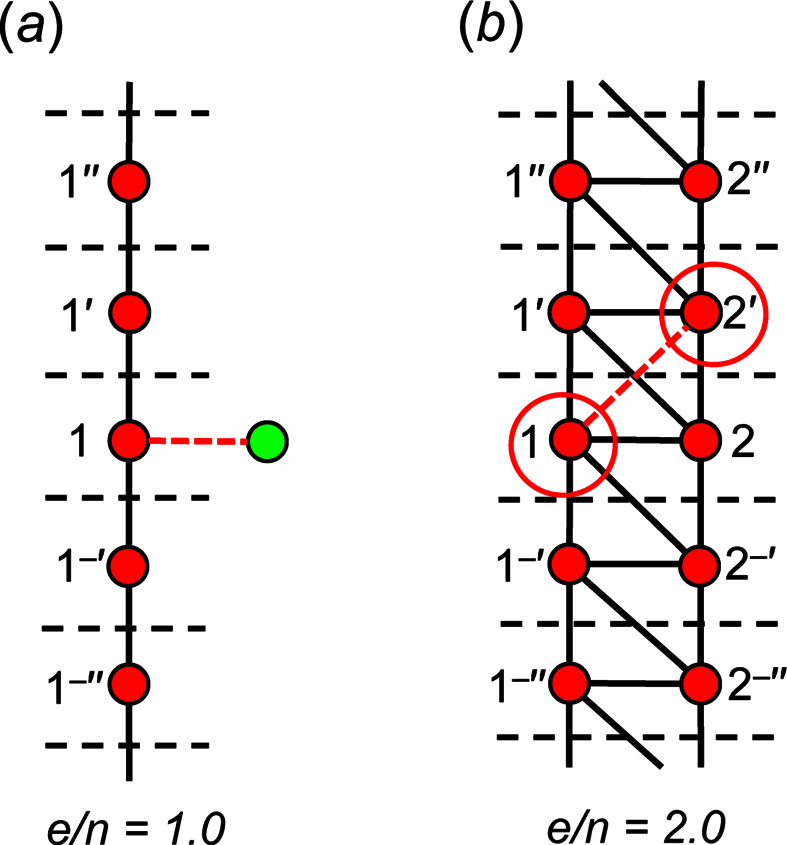
(*a*) A ^2^ *V*_1_ chain graph with *e*/*n* = 1.0. One may attempt to decrease *e*/*n* by adding the green vertex to the chain graph in (*a*), but this requires that an edge (red dashed line) also be added, showing that for any chain graph, *e*/*n* cannot be less than 1.0. (*b*) A ^4^ *V*_2_ chain graph with *e*/*n* = 2.0. One may attempt to increase *e*/*n* by adding an edge (red dashed line) linking vertices 1 and 2. However, this results in 5-connected vertices (shown with red ellipses) which are not allowed, and thus for any chain graph, *e*/*n* cannot exceed 2.0. Legend as in Fig. 10 ▸.

Chain graphs cannot have *e*/*n* < 1 as each vertex must have at least one incident edge and a repeat unit must be connected to two adjacent repeat units by at least one edge along +**c** and −**c** directions. Consider the simplest possible chain graph and the corresponding unit-distance graph in Fig. 16 ▸(*a*) with vertex connectivity ^2^ *V*_1_ and *e*/*n* = 1.0. One may attempt to decrease *e*/*n* by adding a vertex to the topological repeat unit, but this requires that at least one edge is also added to connect that vertex to the chain and thus *e*/*n* remains unchanged at 1.0. Chain graphs cannot have *e*/*n* > 2 as the maximum degree of any vertex is four. Consider the chain graph and the corresponding unit-distance graph in Fig. 16 ▸(*b*) with vertex connectivity ^4^ *V*_2_ and *e*/*n* = 2.0; one may attempt to increase *e*/*n* by adding an edge, but this is not possible as it produces vertices of degree five. One may also attempt to remove a vertex, but this would also remove all edges connected to that vertex. and thus *e*/*n* cannot exceed 2.0.

As shown in Section 4.2.1, all acyclic chain graphs are equalizable and have an equal number of edges and vertices in the repeat unit (*e*/*n* = 1). All acyclic unit-distance graphs have *m*_v_ = *m* and hence are flexible (*m*_v_ = *m*), from which we can conclude that chain graphs with *e*/*n* = 1 are flexible. In cyclic chain graphs, the number of edges must be greater than the number of vertices (*e*/*n* > 1) in the repeat unit. It follows that any acyclic chain graph (*e*/*n* = 1.0) may be converted to a cyclic chain graph by adding one or more edges and in turn, increasing *e*/*n*.

## 7. Compatibility of chain graphs with the metrics of observed chain structures

Thus far, we have shown how embedding a chain graph and equalizing the *T*–*T* distances (edge lengths) of any acyclic chain graph does not restrain the geometry of the corresponding unit-distance graph. We have also shown how equalizing the *T*–*T* distances of a cyclic chain graph may restrain the geometry of the corresponding unit-distance graph, and the degree to which the geometry is restrained is a function of the rigidity of the unit-distance graph (Section 6). However, to fully evaluate the compatibility of any chain graph with the average observed metrics of chains of (*T*O_4_)^*n*−^ tetrahedra in crystals, chains must be embedded in Euclidean space while restraining not only *T*–*T* distances but also *T*⋯*T* separations.

### 7.1. Restraining *T*⋯*T* separations during embedding

**Figure 17:**
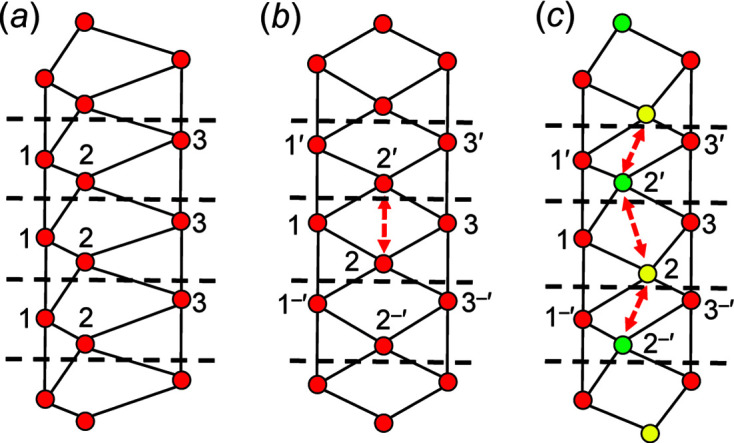
(*a*) A planar geometric graph with unequal edge lengths; (*b*) the unit-distance graph from (*a*) now equalized. In (*b*), the *T*⋯*T* separation 2–2′ is the same length as the *T*–*T* distance and thus this chain geometry is not allowed. (*c*) The unit-distance graph in (*b*) where green vertices are moved out of the plane of the page and yellow vertices are moved in the plane of the page such that the *T*⋯*T* separation 2–2′ is greater than the *T*–*T* distance. Legend as in Fig. 10 ▸.

Consider the chain graph in Fig. 17 ▸(*a*); equalizing the *T*–*T* distances in 2D produces the unit-distance graph in Fig. 17 ▸(*b*) but also produces a *T*⋯*T* separation between vertices 2 and 2′ (and 2 and 2^–^′) that is approximately equal to the *T*–*T* distance. As described in Section 4.3, *T*⋯*T* separations must be at least 1.16 times larger than any *T*–*T* distance; if this is not the case, O^2−^ ions of each tetrahedra will be too close together, forming an unstable arrangement. It follows that a unit-distance graph with the geometry shown in Fig. 17 ▸(*b*) will not occur as a chain of tetrahedra. However, this unit-distance graph is *semi-flexible* in 3D, and we may attempt to lengthen the 2–2′ separation by moving the green vertices out of the plane of the page and the yellow vertices into the plane of the page to produce the unit-distance graph in Fig. 17 ▸(*c*). This will lengthen the 2⋯2′ separation but it is not clear if the 2⋯2′ separation can be increased to 1.16 times the *T*–*T* distance without forcing *T*–*T* distances to vary by more than the allowed limit (∼5%, see Section 4.1.1). How to determine if a chain graph may be embedded in Euclidean space such that it corresponds to a unit-distance graph in which *T*–*T* and *T*⋯*T* restraints are satisfied is described in the following section.

### 7.2. The embedding process

To embed chain graphs in Euclidean space while restraining *T*–*T* distances and *T*⋯*T* separations (as described in Section 4), one must restrain *T*–*T* distances and *T*⋯*T* separations to values observed experimentally in chains of tetrahedra (Section 4.1.1) as follows:

(i) **T*–*T* distances:* the distances between linked vertices are restrained by treating edges as notional springs that behave according to Hooke’s law, 

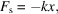

where *F*_s_ is the spring force, *k* is the spring constant (stiffness) and *x* is the spring displacement. Here, the equilibrium spring length represents the ideal *T*–*T* distance observed in chains of tetrahedra (3.06±0.15 Å, Fig. 3 ▸).

(ii) **T*⋯*T* separations:* the distances between unlinked vertices are restrained by a notional repulsive force between them described by Coulomb’s law,

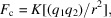

where *F*_c_ is the Coulomb force, *K* is Coulomb’s constant, *q*_1_ and *q*_2_ are the charges associated with each vertex *T*, and *r* is the distance between charges. Coulomb’s constant, *K*, may be adjusted to increase or decrease *F*_c_.

### 7.3. Embedding software: *Graph* *T*–*T*

A detailed description of *Graph* *T*–*T* (V1.0Beta) and the 3D spring-force-directed algorithms used to embed graphs is provided by Day *et al.* (2024 ▸) and only a brief description of the program is given here.

*Graph* *T*–*T* (V1.0Beta) was written to implement the embedding procedure described in Section 7.2. This program uses a 3D spring-force algorithm in which the equilibrium spring length (ideal *T*–*T* distance) can be set to any value (*e.g.* 〈*T*–*T*〉 = 3.06±0.15 Å). For *F*_s_ and *F*_c_ calculations, *k* and *K* are adjustable to allow for deviation of *T*–*T* distances from the set spring length and *T*⋯*T* separations less than the threshold value of γ (less than 1.16 times the *T*–*T* distance).

The net forces acting on vertices of a particular input graph are calculated iteratively according to Newton’s laws of motion using a Barnes–Hut (*N*-body) simulation (Barnes & Hut, 1986 ▸). To avoid computation issues related to the *N*-body problem, the Barnes–Hut simulation groups adjacent vertices as *bodies* and calculates the position of the centre of charge of each body. The net repulsive and attractive forces exerted on all other bodies from the centre of charge of each body are calculated and used to calculate a new position for each vertex after each iteration.

Adjustable embedding parameters in *Graph* *T*–*T* include spring coefficient (*k*), Coulomb’s constant (*K*), spring length, drag coefficient, theta, time step and cooldown time, all of which are described in detail by Day *et al.* (2024 ▸). The embedding parameters used in Tables 1 ▸ ▸ ▸–4 ▸ are referred to as *recommended embedding parameters* by Day *et al.* (2024 ▸). These parameters were determined by embedding a series of chain graphs and iteratively refining each embedding parameter based on agreement of the *T*–*T* distances with the set spring length and increase in the *T*⋯*T* separations. A particular chain graph may be embedded using different sets of embedding parameters to produce geometrically different unit-distance graphs that satisfy the *T*–*T* and *T*⋯*T* restraints; we report only those produced using the variables listed in Tables 1 ▸ ▸ ▸–4 ▸. To ensure that the geometry of unit-distance graphs produced with *Graph* *T*–*T* is easily understood and visually comprehensible, the spring length (and thus the *T*–*T* and *T*⋯*T* restraints) are scaled by a factor of 16.34 (*e.g.* 3.06 × 16.34 = 50.00 Å) and γ = 50 × 1.16 = 58 Å. After a chain graph has been embedded, they may be re-scaled (*e.g.* 50/16.34 = 3.06 Å and 58/16.34 = 3.55 Å) such that they can be compared with *T*–*T* distances and *T*⋯*T* separations observed in crystal structures.

While embedding some chain graphs (particularly chain graphs with a high average vertex connectivity), vertices may become trapped at non-ideal positions (a *false minimum*) with respect to the *T*–*T* distances and *T*⋯*T* separations associated with those vertices. Any unit-distance graph that contains one or more vertices that occupy a false minimum are referred to as *metastable*. Vertices occupying false-minimum positions are referred to as *trapped*, as a temporary increase in the corresponding *T*–*T* distances and *T*⋯*T* separations (*F*_s_ and *F*_c_) towards less ideal values is required for that vertex to move to a more ideal position with respect to the ideal *T*–*T* distances and *T*⋯*T* separations [*e.g.* Fig. 2 from Day *et al.* (2024 ▸)]. To minimize the probability of generating metastable unit-distance graphs, a two-step embedding procedure is used where for the first 15 s of the embedding process, a set of *default embedding parameters* [defined by Day *et al.* (2024 ▸)] is used with *k* set to a relatively small value such that *F*_c_ is sufficiently strong to counteract *F*_s_ and move vertices away from false-minimum positions. For most unit-distance graphs, the positions of the vertices are close to ideal after the first phase of embedding is complete and are then refined once the user-specified embedding parameters are applied in the second phase of embedding. A more detailed description of metastable unit-distance graphs and the two-step embedding process is given by Day *et al.* (2024 ▸).

#### 7.3.1. Unit-distance graph convergence

During the first 1–2 s of the embedding process, all vertices occupy approximately the same position and the *T*–*T* distances and *T*⋯*T* separations are close to zero. As embedding starts, vertices will begin to move apart from one another and as embedding progresses, *T*–*T* distances will approach the set spring length and *T*⋯*T* separations will increase due to mutual repulsion. The *T*⋯*T* separations will increase until *T*–*T* distances are required to deviate (beyond the allowed limit) from the set spring length. At this point, the unit-distance graph has *converged*. *Graph* *T*–*T* calculates and reports the minimum, maximum and average *T*–*T* distances and *T*⋯*T* separations after each iteration such that the user can determine if the unit-distance graph has converged. Once convergence has occurred, vertices will continue to respond to *F*_s_ and *F*_c_ and, in response, will oscillate around a central point in response to these forces. Therefore, average *T*–*T* distances and minimum *T*⋯*T* separations are reported as ranges (R〈*T*–*T*〉 and R〈*T*⋯*T*〉_min_ in Tables 1 ▸ ▸ ▸–4 ▸) and are referred to as the *compatibility parameters* of the unit-distance graph to which they correspond.

Once a given chain graph has been embedded with *Graph* *T*–*T* and has converged, it can be described as:

(i) *Compatible*: if a unit-distance graph converges such that the *T*–*T* and *T*⋯*T* restraints are satisfied, the corresponding chain graph may correspond to a chain of tetrahedra and is considered as potentially *compatible* with a crystal structure.

(ii) *Incompatible*: if a unit-distance graph converges such that the *T*–*T* and/or *T*⋯*T* restraints are not satisfied, the corresponding chain graph cannot correspond to a chain of tetrahedra and is considered *incompatible* with a crystal structure.

## 8. Discussion: testing the compatibility of chain graphs

Day & Hawthorne (2020 ▸) noted several trends regarding the topological properties of chain graphs and the relative abundances of the minerals in which they occur. They made the following observations:

(i) Of the 450 chain-silicate minerals, 375 correspond to 4 non-isomorphic graphs, whereas the other 75 minerals correspond to 46 graphs. Why?

(ii) Why is the abundance of chain-silicate minerals relatively high when O:*T* = 3.00–2.75?

(iii) Why are no chains observed with O:*T* = 2.5–2.0 despite being topologically possible?

(iv) Are there specific topological and/or geometrical properties of a chain that influence the abundance of minerals with that chain topology?

(v) To what extent do the composition and structure of the rest of a mineral influence the geometry and topology of the chains to which they link?

To begin to address these questions, we use *Graph* *T*–*T* to gauge the degree to which selected chain graphs are compatible with the observed *T*–*T* distances and *T*⋯*T* separations in chains of tetrahedra.

### 8.1. Geometric compatibility as a function of vertex connectivity and **e*/*n**

First, we will examine how the geometric compatibility of chain graphs varies as a function of vertex connectivity, *e*/*n* ratio and rigidity by using *Graph* *T*–*T* to embed a series of chain graphs [from Appendix *G* in Day & Hawthorne (2022 ▸)] with increasing *e*/*n* (and rigidity) and comparing the 〈*T*–*T*〉 distances and minimum 〈*T*⋯*T*〉 separations of the corresponding unit-distance graphs with the ideal values. The number of *T*⋯*T* separations less than γ (if any), the planarity, cyclicity, polygon type and degree of medium-range modification (*n*_t_:*n*_g_) will be determined. Many chain graphs may be embedded to produce both planar and non-planar unit-distance graphs that are compatible. Thus, in the *planarity* column of Tables 1 ▸ ▸ ▸–4 ▸, a chain graph is indicated as planar (p) if an embedding exists that produces a planar unit-distance graph even if *Graph* *T*–*T* produces a non-planar unit-distance graph. It follows that any chain graph indicated as non-planar (np) cannot be embedded (in any way) to produce a compatible planar unit-distance graph.

For vertex connectivities ^2^ *V*_2_^3^ *V*_2_ and ^3^ *V*_2_^4^ *V*_1_, all possible non-isomorphic chain graphs are embedded to evaluate how geometrical compatibility varies amongst chains with identical connectivity and *e*/*n* ratios. The ^2^ *V*_2_^3^ *V*_2_ and ^3^ *V*_2_^4^ *V*_1_ chain graphs are listed in Tables 2 ▸ and 3 ▸ using their matrix-element combinations as shown in Appendix *G* in Day & Hawthorne (2022 ▸).

#### 8.1.1. Chain graphs with *e*/*n* = 1.0

We begin by embedding a series of acyclic chain graphs with *e*/*n* = 1.0; the resultant compatibility parameters are given in Table 1 ▸. All chain graphs with *e*/*n* = 1.0 are strictly chains (rather than ribbons or tubes), must be acyclic and flexible (*m*_v_ = *m*), and the backbone chain may or may not link to *n*-membered branches. The chain graphs (1)–(6) in Table 1 ▸ are *compatible* and converge to unit-distance graphs with 〈*T*–*T*〉 50.001–50.008 Å and R〈*T*⋯*T*〉_min_ that is larger than γ = 1.16 × 50 = 58 Å. In Table 1 ▸, chain graphs (1)–(3) converge to unit-distance graphs that show the largest 〈*T*–*T*〉 and R〈*T*⋯*T*〉_min_ in accord with the set spring length (50). Chain graphs (1)–(3) occur in pyroxenes (Morimoto, 1989 ▸), terskite (Grice *et al.*, 2015 ▸) and astrophyllite-group minerals (Sokolova *et al.*, 2017 ▸), respectively. Unlike chain graphs (4)–(6), chain graphs (1)–(3) may be embedded to produce planar unit-distance graphs. However, *Graph* *T*–*T* does not require chain graphs (1)–(3) to converge as planar unit-distance graphs to satisfy the *T*–*T* and *T*⋯*T* restraints. The planarity of embedded chain graphs will be discussed in more detail in Section 8.2.

**Figure 18:**
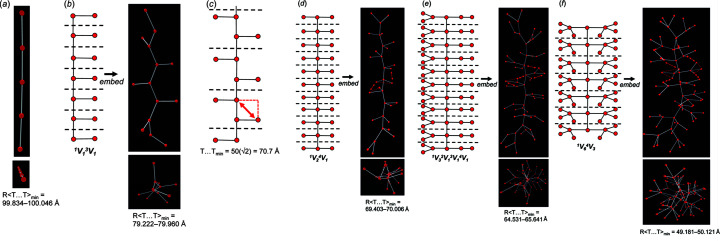
(*a*) The unit-distance graph produced by embedding the ^2^ *V*_1_ chain graph; (*b*) a ^1^ *V*_1_^3^ *V*_1_ chain graph, the corresponding unit-distance graph; (*c*) a planar representation of the unit-distance graph in (*b*) with a straight backbone chain and *T*⋯*T*_min_ = 70.7 Å defined by the square shown with red dashed lines; (*d*) a ^1^ *V*_2_^4^ *V*_1_ chain graph and the corresponding unit-distance graph; (*e*) a ^1^ *V*_3_^2^ *V*_1_^3^ *V*_1_^4^ *V*_1_ chain graph and the corresponding unit-distance graph; and (*f*) a ^1^ *V*_6_^4^ *V*_3_ chain graph and the corresponding unit-distance graph. Each unit-distance graph is shown orthogonal to and along the axis of chain elongation. Red arrows show the *T*⋯*T*_min_ separations and dashed black lines show the repeat unit of each chain graph.

The chain graph ^2^ *V*_1_ [Fig. 18 ▸(*a*)] converges to a straight (planar) unit-distance graph with R〈*T*⋯*T*〉_min_ = 99.834–100.046 Å. The chain graph ^1^ *V*_1_^3^ *V*_1_ in astrophyllite [Fig. 18 ▸(*b*)] is kinked (*n*_g_ > *n*_t_) to maximize the *T*⋯*T* separation between the ^1^ *V*_1_ branches and vertices of the backbone chain. If this chain were straight, the minimum *T*⋯*T* separation would be significantly shorter (∼70.7 Å) as shown in Fig. 18 ▸(*c*). In chain graphs (4), (5) and (6) (Table 1 ▸), branches involve 2, 3 and 5 vertices, respectively, as distinct from 1 vertex in chains (1)–(3). Consequently, there is less space around the backbone chain for the vertices of each branch, and R〈*T*⋯*T*〉_min_ is shorter in the unit-distance graphs that correspond to chain graphs (4)–(6). In the unit-distance graph that corresponds to chain graph (4) [Fig. 18 ▸(*d*)], the two ^1^ *V*_1_ vertices that comprise each branch are forced into closer proximity (shown with red dashed lines) but the *T*⋯*T* separation between vertices of adjacent branches (shown with the red arrow) does not fall below γ. Note that this *T*⋯*T* separation between vertices of adjacent branches is also increased by kinking of the backbone chain. The branches in chain graph (6) [Fig. 18 ▸(*e*)] contain 5 vertices and R〈*T*⋯*T*〉_min_ is shorter than in the unit-distance graphs that correspond to chain graphs (1)–(5). The branches in the unit-distance graph that correspond to chain graph (7) [Fig. 18 ▸(*f*)] contain 8 vertices and R〈*T*⋯*T*〉_min_ is less than γ; this unit-distance graph is *incompatible* and cannot correspond to a chain of tetrahedra as there are four distinct *T*⋯*T* separations in every repeat unit that are less than γ (Table 1 ▸).

We note that for most acyclic chains with *e*/*n* = 1.0, R〈*T*⋯*T*〉_min_ decreases as the number of vertices in the repeat unit (the number of vertices comprising each branch) increases. Despite their flexibility, acyclic chain graphs for which *n*_t_ is relatively large may not be compatible. However, there are a handful of exceptions, such as acyclic chain graphs with ^1^ *V*_1_^2^ *V*_*r*_ branches where *r* = 1 − ∞.

#### 8.1.2. Chain graphs with vertex connectivity ^2^ *V*_2_^3^ *V*_2_, *e*/*n* = 1.25

**Figure 19:**
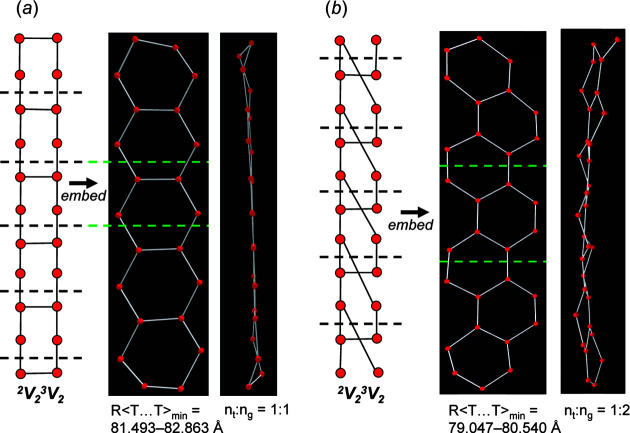
(*a*) The ^2^ *V*_2_^3^ *V*_2_ (10×**1**) **d** chain graph and the corresponding unit-distance graph in which *n*_t_:*n*_g_ = 1:1; (*b*) a ^2^ *V*_2_^3^ *V*_2_, (6×**1**, 2×**2^1^**) **b** chain graph and the corresponding unit-distance graph in which *n*_t_:*n*_g_ = 1:2. Green dashed lines show the repeat unit of each unit-distance graph. Legend as in Fig. 18 ▸.

Here, we embed all non-isomorphic chain graphs with vertex connectivity ^2^ *V*_2_^3^ *V*_2_ and *e*/*n* = 1.25. All ^2^ *V*_2_^3^ *V*_2_ chain graphs are compatible and the compatibility parameters are given in Table 2 ▸. As *e*/*n* > 1.0, all chain graphs are cyclic, and the degree of rigidity is variable. Most ^2^ *V*_2_^3^ *V*_2_ chains show some degree of medium-range modification (Section 6.4) where *n*_g_ > *n*_t_ and embedding forces a non-planar arrangement. There are only five chain graphs (Table 2 ▸) with *n*_t_:*n*_g_ = 1:1 which can be embedded to produce planar unit-distance graphs. Most chain graphs with *n*_t_:*n*_g_ = 1:1 are relatively rigid as they contain 3- and 4-membered polygons (Sections 6.2 and 6.3); the exception is chain graph (10×**1**) **d** [Fig. 19 ▸(*a*)] which corresponds to the chain in amphibole-supergroup minerals (Hawthorne *et al.*, 2012 ▸). Chain graphs are labelled using the matrix-element combinations [*i.e.* (10×**1**) **d**] as described by Day & Hawthorne (2022 ▸, Appendix *G*). The corresponding unit-distance graph has a relatively large R〈*T*⋯*T*〉_min_ (= 81.493–82.863 Å), is *6/18-semi-flexible*, and converges to form a planar arrangement. Chain graph (6×**1**, 2×**2^1^**) **b** [Fig. 19 ▸(*b*)] results in a unit-distance graph that is geometrically similar as it also converges to form a planar arrangement but one in which *n*_t_:*n*_g_ = 1:2. Chain graph (6×**1**, 2×**2^1^**) **b** results in a unit-distance graph that is less flexible and has a slightly shorter R〈*T*⋯*T*〉_min_ (= 79.047–80.540 Å). In general, as *e*/*n* and the rigidity of chain graphs increase, 〈*T*⋯*T*〉_min_ of the corresponding unit-distance graph decreases.

**Figure 20:**
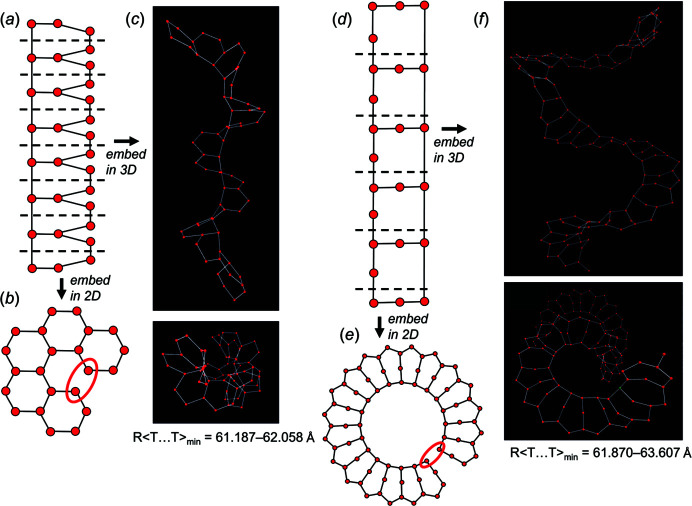
(*a*) The ^2^ *V*_2_^3^ *V*_2_ (8×**1**, 1×**2**) **b** chain graph; (*b*) a unit-distance graph produced by embedding the chain graph in (*a*) in 2D, which forces a *T*⋯*T* separation less than γ (red ellipse); (*c*) a unit-distance graph produced by embedding the chain graph in (*a*) in 3D; (*d*) the ^2^ *V*_2_^3^ *V*_2_, (4×**1**, 1×**2**, 2×**2^1^**) **b** chain graph; (*e*) a unit-distance graph produced by embedding the chain graph in (*d*) in 2D, which forces a *T*⋯*T* separation less than γ (red ellipse); (*f*) a unit-distance graph produced by embedding the chain graph in (*d*) in 3D. Legend as in Fig. 18 ▸.

Consider the chain graph (8×**1**, 1×**2**) **b** [Fig. 20 ▸(*a*), Table 2 ▸). Embedding this chain in 2D forces the corresponding unit-distance graph to curve in on itself and form a ring, a planar arrangement that is incompatible as it forces unrealistic *T*⋯*T* separations [shown with a red ellipse in Fig. 20 ▸(*b*)]. However, embedding this chain in 3D using *Graph* *T*–*T* results in a unit-distance graph that is forced into a helical arrangement that is compatible [Fig. 20 ▸(*c*)]. Consider the chain graph (4×**1**, 1×**2**, 2×**2^1^**) **b** [Fig. 20 ▸(*d*), Table 2 ▸]. When embedded in 2D, the corresponding unit-distance graph is also forced to form a planar ring that is incompatible [Fig. 20 ▸(*e*)]; when embedded in 3D, the unit-distance graph forms a helix. Both chain graphs (8×**1**, 1×**2**) **b** and (4×**1**, 1×**2**, 2×**2^1^**) **b** [Figs. 20 ▸(*c*), 20 ▸(*f*)] result in unit-distance graphs that show a relatively large degree of medium-range modification with *n*_t_:*n*_g_ = 1:12 and 1:20, respectively (Table 2 ▸).

#### 8.1.3. Chain graphs with vertex connectivity ^3^ *V*_2_^4^ *V*_1_, *e*/*n* = 1.67

**Figure 21:**
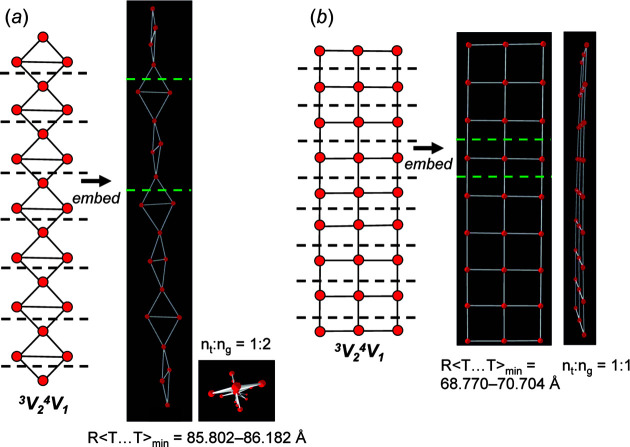
(*a*) The ^3^ *V*_2_^4^ *V*_1_ (2×**1**, 4×**2^1^**) **a** chain graph and the corresponding unit-distance graph in which *n*_t_:*n*_g_ = 1:2; (*b*) the ^3^ *V*_2_^4^ *V*_1_ (4×**1**, 3×**2**) chain graph and the corresponding unit-distance graph in which *n*_t_:*n*_g_ = 1:1. Green dashed lines show the repeat unit of each unit-distance graph. Legend as in Fig. 18 ▸.

Here, we embed all non-isomorphic chain graphs with vertex connectivity ^3^ *V*_2_^4^ *V*_1_ and with *e*/*n* = 1.67. Of the 14 non-isomorphic ^3^ *V*_2_^4^ *V*_1_ chain graphs, nine are incompatible as the corresponding unit-distance graphs have R〈*T*⋯*T*〉_min_ less than γ; the compatibility parameters for all chains are given in Table 3 ▸. All ^3^ *V*_2_^4^ *V*_1_ chain graphs are cyclic and result in unit-distance graphs that show a relatively high degree of rigidity compared with chains with *e*/*n* = 1.0 (Table 1 ▸) and 1.25 (Table 2 ▸). As the degree of rigidity increases, 〈*T*⋯*T*〉 decreases, as is seen by comparing R〈*T*⋯*T*〉_min_ in Tables 2 ▸ and 3 ▸. However, this is not always the case. For example, chain graph (2×**1**, 4×**2^1^**) **a** consists of edge- and vertex-sharing triangles and results in a unit-distance graph that is *6/10-semi-rigid* and has the largest R〈*T*⋯*T*〉_min_ (= 85.802–86.182 Å) of all ^3^ *V*_2_^4^ *V*_1_ chain graphs [Fig. 21 ▸(*a*)]. Chain graph (4×**1**, 3×**2**) consists of edge-sharing squares and results in a unit-distance graph that is *5/10-semi-rigid* (more flexible) but has a shorter R〈*T*⋯*T*〉_min_ (= 68.770–70.704 Å) than chain graph (2×**1**, 4×**2^1^**) **a** as 〈*T*⋯*T*〉_min_ in the corresponding unit-distance graph is restrained across the diagonal of each square (〈*T*⋯*T*〉_min_ = 50 × 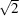 = 70.7) [Fig. 21 ▸(*b*)]. In this case, it is the type of polygon that controls 〈*T*⋯*T*〉_min_ rather than the rigidity of the unit-distance graph.

**Figure 22:**
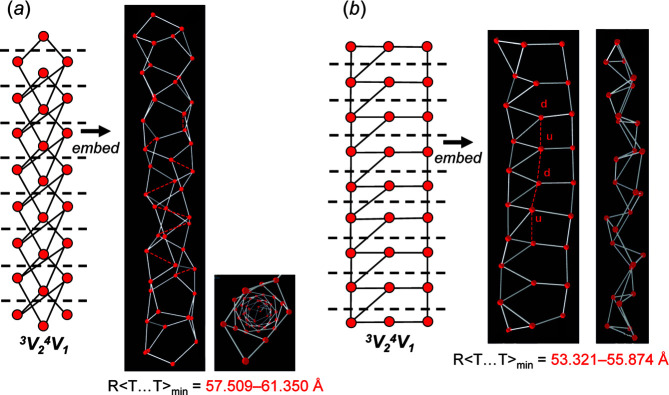
(*a*) The ^3^ *V*_2_^4^ *V*_1_ (2×**1**, 4×**2^2^**) **b** chain graph and the corresponding unit-distance graph; (*b*) the ^3^ *V*_2_^4^ *V*_1_ (2×**1**, 2×**2**, 2×**2^1^**) chain graph and the corresponding unit-distance graph in which vertices are forced into and out of the plane of the unit-distance graph (indicated by a red ‘u’ or ‘d’). Both unit-distance graphs are incompatible and *T*⋯*T*_min_ separations less than γ are shown with red dashed lines. Legend as in Fig. 18 ▸.

Consider the incompatible chain graph (2×**1**, 4×**2^2^**) **b** in Fig. 22 ▸(*a*). This chain is tubular and consists of edge-sharing squares and hexagons, and corresponds to a unit-distance graph that shows medium-range modification (*n*_t_:*n*_g_ = 1:5) and *n*_g_ contains eight *T*⋯*T* separations less than γ. However, this unit-distance graph may or may not be compatible as near-convergence results in R〈*T*⋯*T*〉_min_ = 57.509–61.350 Å that fluctuates above and below γ. However, R〈*T*–*T*〉 = 50.000–50.006 Å and it is likely that *T*–*T* distances may slightly lengthen to increase R〈*T*⋯*T*〉_min_ beyond γ. Consider chain graph (2×**1**, 2×**2**, 2×**2^1^**) [Fig. 22 ▸(*b*)], a ribbon of edge-sharing triangles and pentagons; in the corresponding unit-distance graph, *n*_g_ contains two *T*⋯*T* separations less than γ (R〈*T*⋯*T*〉_min_ = 53.321–55.874 Å), and this chain graph is incompatible. In Fig. 22 ▸(*b*), note how alternating ^3^ *V*_1_ vertices are forced into and out of the plane of the page (indicated by ‘u’ and ‘d’ vertex labels) to increase the *T*⋯*T* separation between these vertices (shown with red dashed lines); this results in medium-range modification (*n*_t_:*n*_g_ = 1:2).

#### 8.1.4. Chain graphs with *e*/*n* = 2.0

Here, we embed a series of chain graphs with *e*/*n* = 2.0, and the resultant compatibility parameters are given in Table 4 ▸. All chain graphs with *e*/*n* = 2.0 must be cyclic, must contain only ^4^ *V_r_* vertices, and must be ribbons and tubes rather than chains. In Table 4 ▸, incompatible chain graphs result in unit-distance graphs with shorter R〈*T*⋯*T*〉_min_ values than ^3^ *V*_2_^4^ *V*_1_ chain graphs (Table 3 ▸) and are semi-rigid to rigid, containing 3- and 4-membered polygons.

**Figure 23:**
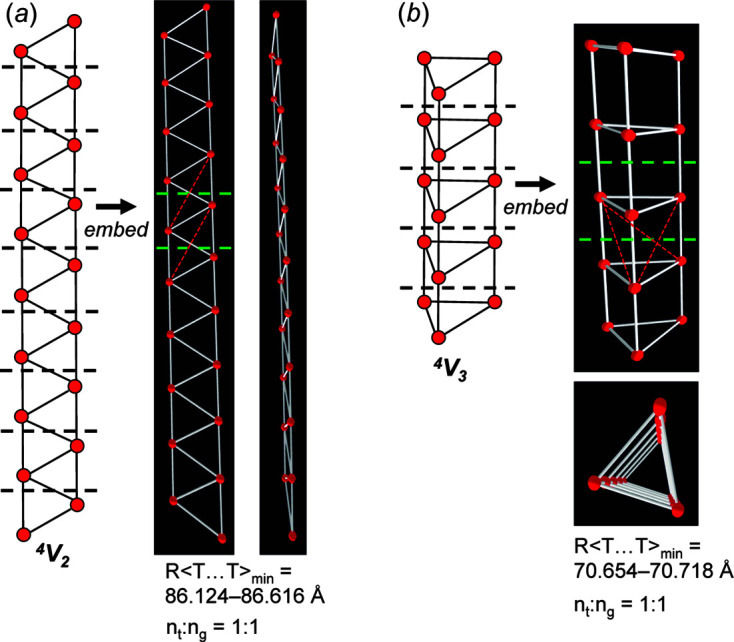
(*a*) A ^4^ *V*_2_ chain graph and the corresponding unit-distance graph in which *n*_t_:*n*_g_ = 1:1; (*b*) a ^4^ *V*_3_ chain graph and the corresponding unit-distance graph in which *n*_t_:*n*_g_ = 1:1. The *T*⋯*T*_min_ separations are shown with red dashed lines. Legend as in Fig. 18 ▸.

Relative to chain graphs with *e*/*n* = 1.0, 〈*T*⋯*T*〉_min_ decreases for embedded chain graphs with *e*/*n* = 2.0 as the number of vertices in *n*_t_ increases, and most compatible chain graphs with *e*/*n* = 2.0 have a small number of vertices in *n*_t_ (2–4). Consider chain graph (1) (Table 4 ▸) in Fig. 23 ▸(*a*) [isomorphic with the chain in Fig. 15 ▸(*a*)]. This chain graph consists of edge-sharing triangles and the corresponding unit-distance graph is *3/4-semi-rigid*. Here, R〈*T*⋯*T*〉_min_ = 86.124–86.616 Å, which is relatively large as both vertices in *n*_t_ are connected to each other and thus *T*⋯*T*_min_ (shown with red dashed lines) extends outside *n*_g_. As shown in Fig. 15 ▸, this chain graph may also converge to produce non-planar chains, but this would force a decrease in R〈*T*⋯*T*〉_min_ and cannot be produced using *Graph* *T*–*T*. Chain graph (2) [Fig. 23 ▸(*b*), Table 4 ▸] is non-planar (tubular), consists of edge-sharing triangles and squares, and the corresponding unit-distance graph is *1/3-semi-flexible* but has a shorter R〈*T*⋯*T*〉_min_ (= 70.654–70.718 Å) than the chain graph in Fig. 23 ▸(*a*). Like the ^3^ *V*_2_^4^ *V*_1_ chain graph in Fig. 21 ▸(*b*), 〈*T*⋯*T*〉_min_ is restrained across the diagonal of each square [shown by red dashed lines in Fig. 23 ▸(*b*)]. The chain graphs in Figs. 21 ▸(*b*) and 23 ▸(*b*) are both examples of how R〈*T*⋯*T*〉_min_ is controlled not only by the rigidity of the corresponding unit-distance graph but also by the type of polygon comprising the chain graph.

**Figure 24:**
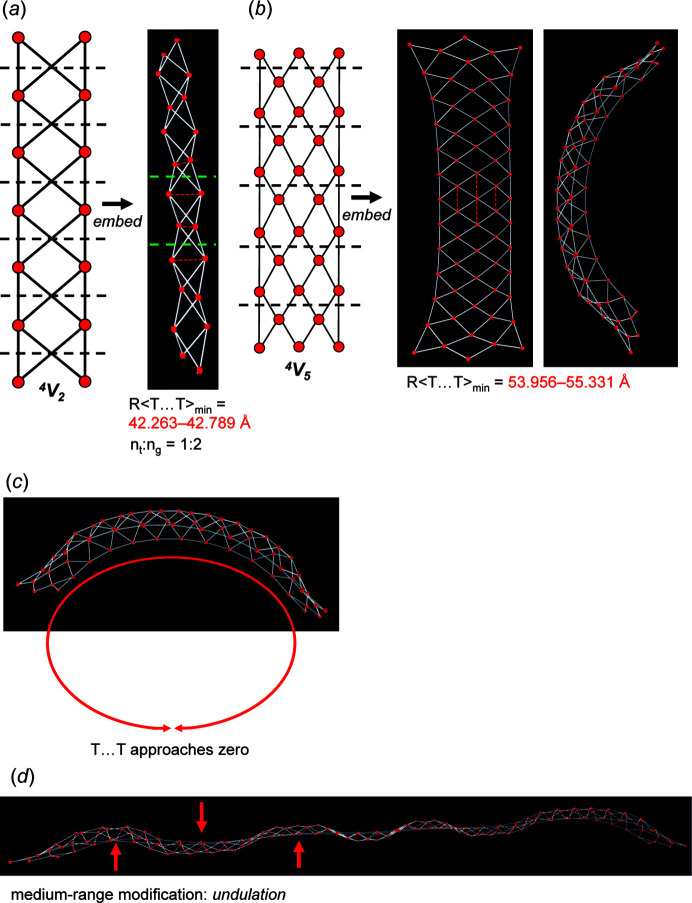
(*a*) A ^4^ *V*_2_ chain graph and the corresponding unit-distance graph in which *n*_t_:*n*_g_ = 1:2; (*b*) a ^4^ *V*_5_ chain graph and the corresponding unit-distance graph. Both unit-distance graphs are incompatible and the *T*⋯*T*_min_ separations less than γ are shown with red dashed lines. (*c*) The unit-distance graph in (*b*) with a red arrow showing how medium-range modification will force *T*⋯*T* separations to approach zero as the length of the chain increases. (*d*) The unit-distance graph in (*b*) in which the length of the chain has been increased to show how a modulated geometry (shown with red arrows) is required to prevent *T*⋯*T* separations from approaching zero as shown in (*c*). Legend as in Fig. 18 ▸.

In Section 4.2.2, we show that the ^4^ *V*_2_ chain graph in Fig. 7 ▸ cannot be equalized to produce a unit-distance graph without forcing the 1–2 separation to become zero. However, embedding this chain graph [graph (6), Table 4 ▸] using *Graph* *T*–*T* results in a unit-distance graph [Fig. 24 ▸(*a*)] in which every second repeat unit is rotated 90° such that *n*_t_:*n*_g_ = 1:2 and R〈*T*⋯*T*〉_min_ = 42.263–42.789 Å. Although this chain graph is incompatible, it provides an example of the degree to which medium-range modification (*n*_g_ > *n*_t_) can increase 〈*T*⋯*T*〉_min_. Chain graph (3) [Fig. 24 ▸(*b*)] is incompatible and provides an extreme example of medium-range modification in which the corresponding unit-distance graph is forced to curve along both the long and short axes of the chain. Increasing the length of this unit-distance graph will force the ends of the chain to close in on each other, resulting in *T*⋯*T* separations that approach zero [Fig. 24 ▸(*c*)]. To prevent this, the direction of curvature must alternate along the length of the chain to produce a modulated unit-distance graph [Fig. 24 ▸(*d*)].

### 8.2. Isomorphic observed and embedded chain geometries

By comparing the geometries of unit-distance graphs (embedded using *Graph* *T*–*T*) with isomorphic chains of tetrahedra observed in minerals (or synthetics), we may determine what geometrical properties of chains are due to linkage with the interstitial structure. We may also better understand the modes by which a particular chain (unit-distance graph) will distort (allow deviation of *T*–*T* distances and *T*⋯*T* separations from their ideal values) to accommodate linkage with the interstitial structure (*e.g.* a sheet or ribbon of *M* octahedra).

**Figure 25:**
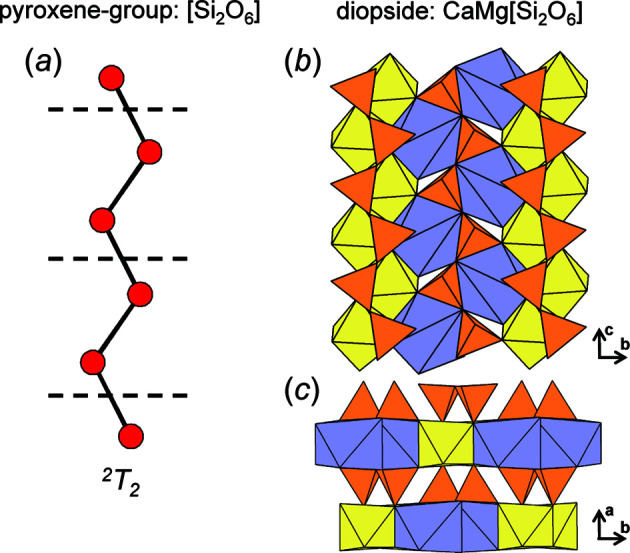
(*a*) A ball-and-stick representation of the ^2^ *T*_2_ chain in pyroxenes. The structure of diopside projected (*b*) along the *c* axis and (*c*) into the *c* axis where Mg octahedra are shown in yellow and Ca polyhedra are shown in blue. Dashed black lines show the geometrical repeat unit of the chain.

**Figure 26:**
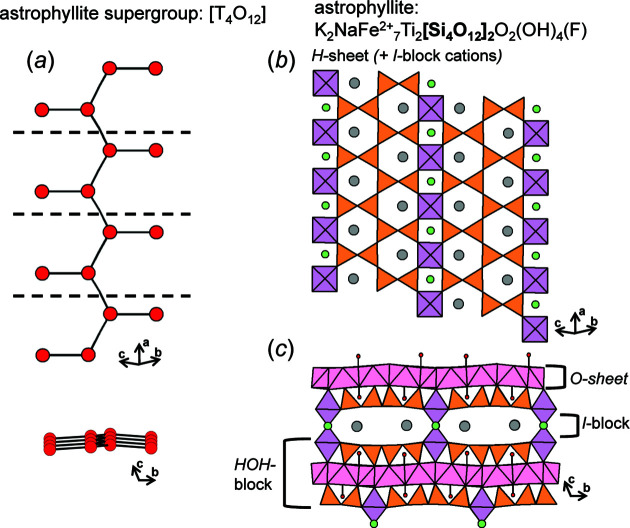
(*a*) A ball-and-stick representation of the ^1^ *T*_2_^3^ *T*_2_ chain in astrophyllite-supergroup minerals and the structure of astrophyllite showing (*b*) the *H* sheet (and *I*-block cations); (*c*) the *HOH* block, *I* block and *O* sheet. The *A* and *B* cations are shown as grey and green circles, respectively. The *D* cations are shown as purple Ti octahedra and the Fe octahedra of the *O* sheet are shown in pink. H atoms are shown as red circles. Dashed black lines show the geometrical repeat unit of the chain.

**Figure 27:**
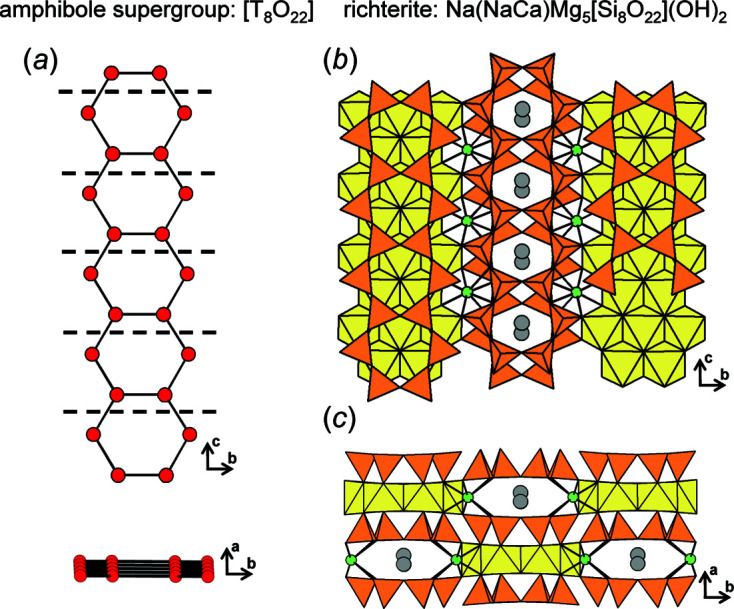
(*a*) A ball-and-stick representation of the ^2^ *T*_2_^3^ *T*_2_ chain in amphibole-supergroup minerals. The structure of richterite projected (*b*) in the a* direction, and (*c*) along the *c* axis where the *M*1–*M*3 cations are shown as yellow Mg octahedra and the *M*4 and *A*2/*A*(*m*) cations are shown as green and grey circles, respectively. Dashed black lines show the geometrical repeat unit of the chain.

Consider the ^2^ *V*_1_ chain graph in Fig. 18 ▸(*a*) which converges to produce a unit-distance graph where *n*_t_:*n*_g_ = 1:1. This ^2^ *V*_1_ chain graph occurs in pyroxene-supergroup minerals as a ^2^ *T*_2_ chain [Fig. 25 ▸(*a*)] in which kinking of the chain is required to facilitate linkage of each Si tetahedron with Mg octahedra [Fig. 25 ▸(*b*)]. The chain graph in Fig. 18 ▸(*a*) converges to a straight (planar) unit-distance graph and thus one cannot assume that the planarity of the ^2^ *T*_2_ chain in diopside is due only to linkage with sheets of ^[7]^Ca polyhedra and Mg octahedra [Fig. 25 ▸(*c*)]. The relation between the geometry of ^2^ *T_r_* chains (*n*_g_) and the size of cations comprising the interstitial structure of pyroxenes and pyroxenoids has been described in detail (Belov, 1961 ▸; Takéuchi *et al.*, 1976 ▸; Nagashima & Armbruster, 2010 ▸; Thompson *et al.*, 2016 ▸). However, for specific chain graphs, kinking is also produced by embedding while restraining *T*–*T* distances and *T*⋯*T* separations. Consider the ^1^ *V*_1_^3^ *V*_1_ chain graph in Fig. 18 ▸(*b*) which converges to produce a non-planar unit-distance graph. Here, ^1^ *V*_1_ branches extend in 3D and the backbone chain is kinked to maximize 〈*T*⋯*T*〉_min_. This ^1^ *V*_1_^3^ *V*_1_ chain graph occurs in astrophyllite-supergroup minerals as a planar ^1^ *T*_2_^3^ *T*_2_ chain [Fig. 26 ▸(*a*)] and we may assume that linkage of this chain to *M* octahedra of the planar *O sheet* in astrophyllite [Fig. 26 ▸(*c*)] requires the chain to be planar, a geometry significantly different from that in Fig. 18 ▸(*b*). In astrophyllite, the backbone of the ^1^ *T*_2_^3^ *T*_2_ chain is kinked to accommodate linkage to polyhedra of the *O sheet* and *I block* [Fig. 26 ▸(*b*)]. However, such kinking is also observed in Fig. 18 ▸(*b*) and thus one cannot assume such kinking is due solely to linkage with the interstitial structure in astrophyllite. Consider the ^2^ *V*_2_^3^ *V*_2_ chain graph in Fig. 19 ▸(*a*) which converges to produce a planar unit-distance graph with *n*_t_:*n*_g_ = 1:1. This ^2^ *V*_2_^3^ *V*_2_ chain graph occurs in amphibole-supergroup minerals as a ^2^ *T*_2_^3^ *T*_2_ ribbon of edge-sharing hexagons [Fig. 27 ▸(*a*)] that also may be viewed as two planar ^2^ *T*_2_ pyroxene-type backbone chains that are kinked to accommodate linkage to a planar ribbon of Mg^2+^ octahedra [Figs. 27 ▸(*b*), 27 ▸(*c*)]. Note that the geometry (*e.g.* planarity and kinking) of the converged ^2^ *V*_2_^3^ *V*_2_ unit-distance graph [Fig. 19 ▸(*a*)] and the geometry of the ^2^ *T*_2_^3^ *T*_2_ ribbon [Fig. 27 ▸(*a*)] in amphiboles are identical. It follows that the geometry of the ribbon of tetrahedra in amphiboles may be solely due to the intrinsic topological and geometrical properties of the ribbon rather than due to linkage with the ribbons of octahedra.

As shown by Day & Hawthorne (2020 ▸), the most abundant chain-silicate structures are layered and consist of planar chains or ribbons of tetrahedra linked to planar sheets or ribbons of larger lower-valence cations of higher coordination (usually octahedral). However, most chain graphs converge as non-planar arrangements [*e.g.* Figs. 18 ▸(*b*), 21 ▸(*a*), 22 ▸(*b*), 24 ▸(*a*) and 24 ▸(*b*)] and thus must distort to accommodate linkage to a planar module of the interstitial structure. Notable exceptions are the ^2^ *V*_1_ and ^2^ *V*_2_^3^ *V*_2_ chain graphs in amphiboles and pyroxenes, which converge to produce planar unit-distance graphs. The degree to which a given chain graph may distort to accommodate linkage to a planar structure is a function of its rigidity and *e*/*n* ratio.

#### 8.2.1. Observed trends in compatibility parameters

Examination of Tables 2 ▸ ▸–4 ▸ shows that chain graphs embedded to produce unit-distance graphs that show no (or minimal) medium-range distortion (*n*_t_:*n*_g_ = 1:1 or 1:2) are typically planar. As the degree of medium-range distortion increases, chain graphs converge to non-planar unit-distance graphs. All ^2^ *V*_2_^3^ *V*_2_ chain graphs with *e*/*n* = 1.25 (Table 2 ▸) are compatible whereas only five ^3^ *V*_2_^4^ *V*_1_ chain graphs with *e*/*n* = 1.67 (Table 3 ▸) are compatible. Thus, there is likely a point between *e*/*n* = 1.25 and 1.67 where the rigidity of unit-distance graphs reaches a point beyond which no degree of medium-range modification can lengthen 〈*T*⋯*T*〉 separations such that they are greater than γ.

**Figure 28:**
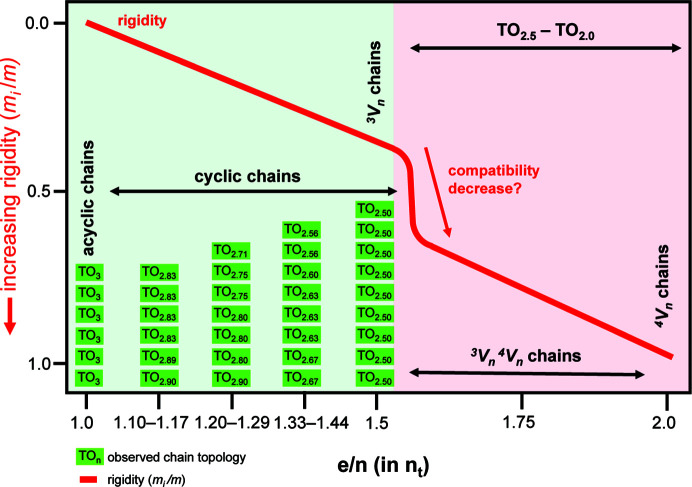
The *e*/*n* ratio of chain graphs with *e*/*n* = 1.0–2.0 as a function of rigidity (*m*_i_/*m*). Each green box represents a unique chain topology observed in minerals. The area shaded in pale green represents the range in *e*/*n* ratio for chains in minerals and the area shaded in pink represents the range in *e*/*n* ratio for chains that are not observed in minerals. Note the sharp increase in rigidity at *e*/*n* = 1.5.

In chain-silicate minerals, the maximum *e*/*n* = 1.5. Day & Hawthorne (2020 ▸) describe 39 minerals that contain ^3^ *V_r_* ribbons and tubes with *e*/*n* = 1.5, including epididymite-, litidionite-, tuhualite- and canasite-group minerals (Pozas *et al.*, 1975 ▸; Chiragov & Shirinova, 2004 ▸; Gatta *et al.*, 2008 ▸; Khomyakov *et al.*, 2009 ▸; Schmidmair *et al.*, 2018 ▸; Merlino & Biagioni, 2018 ▸). There are no minerals or synthetic compounds that contain chains of tetrahedra with *e*/*n* > 1.5, and *e*/*n* = 1.5 may be the threshold at which the compatibility of chain graphs decreases drastically. In Fig. 28 ▸, the rigidity (*m*_i_/*m*, shown as a red line) of observed chain arrangements is shown as a function of their *e*/*n* (in *n*_t_), and the number of non-isomorphic chain arrangements for a given *e*/*n* is indicated using green rectangles. The number of non-isomorphic chains for a given *e*/*n* (or *e*/*n* range) increases from *e*/*n* = 1.0 to 1.5, and *T*O_*n*_ decreases from *T*O_3_ where *e*/*n* = 1.0 to *T*O_2.50_ where *e*/*n* = 1.5. At *e*/*n* = 1.5, all chain arrangements have vertex connectivity ^3^ *V*_*n*_ and are ribbons or tubes (Day & Hawthorne, 2020 ▸). Beyond *e*/*n* = 1.5, there is a sharp increase in rigidity and no chain arrangements in the range 1.5 < *e*/*n* < 2.0 occur in minerals or synthetic compounds. This area (shaded in pink) contains *T*O_2.5_–*T*O_2.0_ chains with 3- and 4-connected tetrahedra. As shown in Sections 8.1.3 and 8.1.4, most chain graphs with *e*/*n* > 1.5 correspond to relatively rigid unit-distance graphs that show *T*⋯*T* separations less than γ and are thus incompatible. The introduction of 4-connected vertices at *e*/*n* > 1.5 results in a significant increase in rigidity and, most likely, a decrease in the compatibility of the corresponding chain arrangements, which may explain the absence of such chains in minerals. Note that in Fig. 28 ▸, the red line is only a general approximation of the trend in rigidity as a function of *e*/*n* as the rigidities for all ∼1500 non-isomorphic chain graphs generated by Day & Hawthorne (2022 ▸) were not calculated. As shown in Section 8.1, rigidity varies also as a function of variables other than *e*/*n*, such as polygon type, and in some cases, a chain with a slightly higher *e*/*n* may be less rigid than a chain with a slightly lower *e*/*n*.

**Figure 29:**
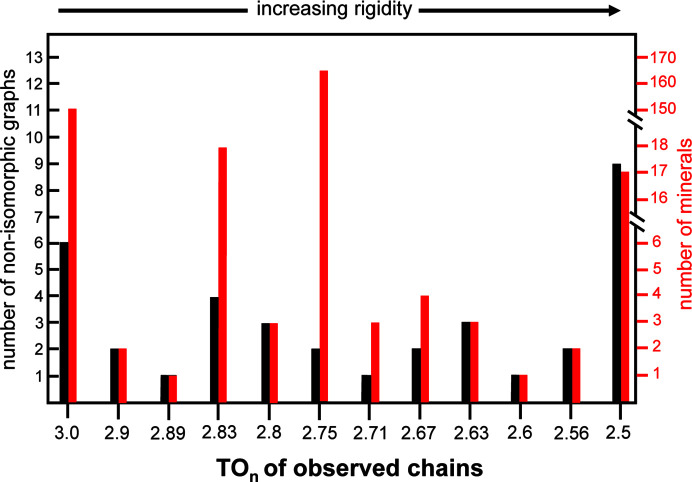
The stoichiometry (*T*O_*n*_) of chains in minerals as a function of the number of non-isomorphic chain graphs and the number of distinct mineral species that correspond to each (*T*O_*n*_), where *n* = 3.0–2.5.

One might expect that, as the rigidity of chain graphs increases, the stability and/or abundance of the corresponding minerals decreases. However, this is not always the case, as shown in Fig. 29 ▸, in which the number of non-isomorphic chain graphs (black lines) and the number of different minerals in which those chains occur (red lines) are plotted for *T*O_3.0_–*T*O_2.5_. The six non-isomorphic *T*O_3_ chain graphs are flexible and correspond to approximately ∼150 different minerals including pyroxenes, pyroxenoids, astrophyllite-supergroup minerals *etc*. The *T*O_2.9_–*T*O_2.8_ chain graphs correspond to 24 different minerals. The *T*O_2.75_ chains, despite being relatively more rigid, correspond to ∼170 different minerals, the majority of which are amphibole-supergroup minerals; thus other properties of chain graphs must affect the stability and abundance of the minerals in which they occur. As discussed in Section 8.1.2, the (10×**1**) **d** chain graph (Table 2 ▸) in amphiboles is one of the few ^2^ *V*_2_^3^ *V*_2_ chains that converges to a planar ribbon which may facilitate efficient linkage to the planar ribbons of *M* octahedra in the amphibole structure (Hawthorne, 1983*b* ▸; Hawthorne & Oberti, 2007 ▸).

## 9. Results and conclusions

This series of papers (Day & Hawthorne, 2020 ▸, 2022 ▸) deals with chains of (*T*O_4_) tetrahedra (*T* = Si^4+^ plus P^5+^, V^5+^, As^5+^, Al^3+^, Fe^3+^, B^3+^, Be^2+^, Zn^2+^ and Mg^2+^) primarily in minerals and attempts to understand the factors affecting the bond topology and geometry of such chains. To understand how the properties of chain graphs influence the incorporation of corresponding chains of tetrahedra into crystal structures, we have examined the possible restraints on embedding chain graphs into Euclidean space. In order to occur in a crystal structure, the metrics of the embedded chain (unit-distance graph) must be congruent with what is observed in crystal structures, specifically *T*–*T* distances approximately equal to the grand 〈*T*–*T*〉 distance (3.06±0.15 Å) in chain-silicate minerals, and *T*⋯*T* approaches that are not shorter than the minimum observed value (3.71 Å) in chain-silicate minerals. If a chain graph can be embedded to produce a unit-distance graph with *T*–*T* distances that are approximately equal, that chain graph is designated as *equalizable*, otherwise it is *non-equalizable*.

We show that equalization of all *acyclic* chain graphs is possible in 2D and 3D, and that equalization of most *cyclic* graphs is possible in 3D but not necessarily in 2D. Non-equalizable chain graphs cannot form chains of tetrahedra that are compatible with crystal structures. To examine why certain cyclic graphs are non-equalizable, we develop a method for calculating the *rigidity* of chain graphs once embedded in Euclidean space to produce a unit-distance graph. Using the vertex subsets of a chain graph, all unique ways in which non-isomorphic vertices may be moved (ways in which the corresponding unit-distance graph may be distorted) are derived and are designated *modes of geometric modification*. If a mode (*m*) is applied to a unit-distance graph such that a new geometrically distinct unit-distance graph is produced without changing the lengths of any edges, the mode is designated as valid (*m*_v_); if a new geometrically distinct unit-distance graph cannot be produced, the mode is invalid (*m*_i_). Rigid unit-distance graphs have *m*_i_ = *m*, flexible unit-distance graphs have *m*_v_ = *m* (*m*_i_ = 0), and the rigidity of unit-distance graphs is a function of *m*_i_/*m*. In general, as the average connectivity of vertices (edge-to-vertex ratio, *e*/*n*) in a chain graph increases, the rigidity (*m*_i_/*m*) of the corresponding unit-distance graph increases.

In some cases, equalization forces unlinked vertices (*T*⋯*T* separations) to be too close, and this is the principal restraint on embedded chain graphs being able to form chains of tetrahedra in crystal structures. During the embedding process, we restrain the minimum *T*⋯*T* separation to 3.71 Å, the minimum *T*⋯*T* separation observed in all chains of tetrahedra in minerals and selected inorganic crystals. A software package, *Graph* *T*–*T*, was developed to embed graphs in Euclidean space while restraining the *T*–*T* distances and *T*⋯*T* separations to realistic values. If the resultant unit-distance graphs have geometries that satisfy these restraints, they are *compatible* with the metrics of crystal structures and may occur in crystal structures; if not, they are *incompatible* with the metrics of crystal structures and will not occur.

Chain graphs with *e*/*n* = 1.0, 1.25, 1.67 and 2.0 [taken from Day & Hawthorne (2022 ▸)] were embedded using the software *Graph* *T*–*T* (Day *et al.*, 2024 ▸). As the average vertex-connectivity increases, the *e*/*n* ratio increases. All chains with *e*/*n* < 1.5 (that were tested) are compatible and many chains with *e*/*n* > 1.5 are incompatible. This compatibility change at *e*/*n* = 1.5 coincides with a marked increase in rigidity and many chains with *e*/*n* ≥ 1.5 require significant distortion (*e.g.* development of non-planar modulated and helical unit-distance graphs) to satisfy the *T*–*T* and *T*⋯*T* restraints. Such chain arrangements may not link efficiently to the typical planar layered interstitial structure that is common in chain-silicate minerals. In addition, *e*/*n* = 1.5 corresponds to the stoichiometry *T*O_2.5_, possibly accounting for why chains of composition *T*O_<2.5_ do not occur in crystals or related synthetic compounds. Chain arrangements with particular connectivities (*e*/*n* < 1.5) correspond to unit-distance graphs that are relatively flexible, and these unit-distance graphs correspond to chains of tetrahedra observed in the most abundant chain-silicate minerals (*e.g.* pyroxenes and amphiboles). In general, as the *e*/*n* ratio increases, the rigidity of the chain increases and the rarity of the mineral in which such arrangements are observed increases. It is abundantly clear that chain arrangements with particular topological and geometrical properties are more favoured during crystallization compared with others, and that such properties affect the abundance of chain-silicate minerals. Future work will focus on other factors that control the relative abundance of silicate minerals such as the topological (connectivity of cations) and geometrical (rigidity and flexibility) properties of the interstitial complex and the geochemical constraints associated with the environment of crystallization that cause particular chemical compositions to be common and others to be rare.

## Figures and Tables

**Table 1:** Compatibility parameters for chain graphs with *e*/*n* = 1.00 Initial *T*–*T* distance (spring length) set to 50 Å, drag coefficient = 0.02, *k* = 0.0035–0.0052, *K* = −0.003, time step = 20 ms, cooldown time = 5 × 10^6^ ms. Theta (*q*_c_) for Barnes–Hut simulation was varied depending on complexity of the input graph. Values calculated after cooldown time has elapsed or until *T*⋯*T* separations have converged. Allowed variation in *T*–*T* = 47.55–52.45 Å, minimum allowable *T*⋯*T* = γ = 58.00 Å. Acy/cy-poly = acyclic/cyclic polygon; c/r/t = chain, ribbon, tube; p = planar; np = non-planar.

Chain graph (*^c^V_r_*)	〈*T*–*T*〉 (Å)	R〈*T*–*T*〉 (Å)	R〈*T*⋯*T*〉_min_ (Å)	*T*⋯*T* < γ	Planarity	Acy/cy-poly	c/r/t	Observed
(1) ^2^ *V*_1_	50.001	50.001–50.001	99.834–100.046	–	p	acy	c	Pyroxenes
(2) ^1^ *V*_1_^2^ *V*_1_^3^ *V*_1_	50.001	50.001–50.001	82.232–83.560	–	p	acy	c	Terskite
(3) ^1^ *V*_1_^3^ *V*_1_	50.002	50.001–50.003	79.222–79.960	–	p	acy	c	Astrophyllite
(4) ^1^ *V*_2_^4^ *V*_1_	50.003	50.002–50.009	69.403–70.006	–	np	acy	c	No
(5) ^1^ *V*_2_^3^ *V*_2_	50.004	50.001–50.005	67.584–68.184	–	np	acy	c	No
(6) ^1^ *V*_3_^2^ *V*_1_^3^ *V*_1_^4^ *V*_1_	50.008	49.156–50.654	64.531–65.641	–	np	acy	c	No
(7) ^1^ *V*_6_^4^ *V*_3_	50.010	48.336–51.424	49.181–50.121	4	np	acy	c	No

**Table 2:** Compatibility parameters for all non-isomorphic ^2^ *V*_2_^3^ *V*_2_ chain graphs (*e*/*n* = 1.25) Initial *T*–*T* distance (spring length) set to 50 Å, drag coefficient = 0.02, *k* = 0.0035–0.0052, *K* = −0.003, time step = 20 ms, cooldown time = 5 × 10^6^ ms. Theta (*q*_c_) for Barnes–Hut simulation was varied depending on complexity of the input graph. Values calculated after cooldown time has elapsed or until *T*⋯*T* separations have converged. Allowed variation in *T*–*T* = 47.55–52.45 Å, minimum allowable *T*⋯*T* = γ = 58.00 Å. Unconnected chain graphs are omitted. Chain graphs labelled using matrix-element combination as shown by Day & Hawthorne (2022 ▸, Appendix *G*). Other abbreviations as given in Table 1 ▸. r (s) indicates a ribbon that forms a spiral or helix upon embedding.

Chain graph	〈*T*–*T*〉 (Å)	R〈*T*–*T*〉 (Å)	R〈*T*⋯*T*〉_min_ (Å)	*T*⋯*T* < γ	Planarity	Acy/cy (poly)	*n*_t_:*n*_g_	c/r/t	Observed
(10×**1**) **k**	50.009	48.384–51.782	81.759–82.864	–	np	cy-10	1:8	t	No
(10×**1**) **d**	50.002	50.001–50.003	81.493–82.863	–	p	cy-6	1:1	r	Amphiboles
(10×**1**) **m**	50.008	48.228–51.897	81.149–82.316	–	np	cy-10	1:8	t	No
(10×**1**) **h**	50.014	48.044–51.340	80.335–81.734	–	np	cy-10	1:4	t	No
(10×**1**) **i**	50.015	48.304–51.704	80.246–81.662	–	np	cy-10	1:4	t	No
(6×**1**, 2×**2^1^**) **c**	50.002	50.001–50.003	79.831–81.349	–	np	cy-10	1:3	t	No
(6×**1**, 2×**2^1^**) **b**	50.002	50.001–50.003	79.047–80.540	–	p	cy-6	1:2	r	No
(4×**1**, 1×**2**, 2×**2^2^**) **b**	50.006	48.284–51.802	79.439–80.429	–	np	cy-8	1:2	r	No
(10×**1**) **n**	50.006	48.255–51.824	79.225–80.134	–	np	cy-10	1:7	t	No
(8×**1**, 1×**2**) **d**	50.005	48.474–51.852	78.224–79.694	–	np	cy-8	1:3	r	No
(8×**1**, 1×**2**) **a**	50.019	48.312–51.756	76.305–78.448	–	p	cy-3	1:1	c	No
(6×**1**, 2×**2^1^**) **d**	50.009	48.273–51.948	77.079–77.907	–	np	cy-10	1:3	t	No
(8×**1**, 1×**2**) **c**	50.003	49.117–51.710	73.273–75.099	–	np	cy-7	1:2	r	No
(10×**1**) **f**	50.008	48.867–51.137	71.369–73.842	–	np	cy-10	1:3	t	No
(10×**1**) **c**	50.002	50.001–50.003	70.897–72.396	–	np	cy-7	1:2	r	No
(6×**1**, 2×**2**)	50.002	50.001–50.004	69.896–71.974	–	np	cy-8	1:2	r	No
(10×**1**) **a**	50.001	49.991–50.021	70.296–71.508	–	p	cy-4	1:1	c	Vlasovite
(6×**1**, 2×**2^1^**) **a**	50.001	50.001–50.002	69.736–70.305	–	p	cy-4	1:1	c	No
(10×**1**) **b**	50.001	50.001–50.001	68.573–69.447	–	p	cy-3	1:1	c	No
(6×**1**, 2×**2^2^**) **a**	50.002	50.001–50.004	66.462–69.381	–	np	cy-8	1:2	r	No
(2×**1**, 2×**2^1^**, 2×**2^2^**)	50.007	48.036–51.725	68.061–69.001	–	np	cy-8	1:2	r	No
(4×**1**, 1×**2**, 2×**2^1^**) **b**	50.007	48.146–51.956	61.870–63.607	–	np	cy-7	1:20	r (s)	No
(8×**1**, 1×**2**) **b**	50.007	48.204–51.837	61.187–62.058	–	np	cy-6	1:12	r (s)	No
(6×**1**, 2×**2^2^**) **c**	50.010	48.326–51.716	60.232–61.831	–	np	cy-10	1:8	t	No

**Table 3:** Compatibility parameters for all non-isomorphic ^3^ *V*_2_^4^ *V*_1_ chain graphs (*e*/*n* = 1.67) Initial *T*–*T* distance (spring length) set to 50 Å, drag coefficient = 0.02, *k* = 0.0035–0.0052, *K* = −0.003, time step = 20 ms, cooldown time = 5 × 10^6^ ms. Theta (*q*_c_) for Barnes–Hut simulation was varied depending on complexity of the input graph. Values calculated after cooldown time has elapsed or until *T*⋯*T* separations have converged. Allowed variation in *T*–*T* = 47.55–52.45 Å, minimum allowable *T*⋯*T* = γ = 58.00 Å. Unconnected chain graphs are omitted. Chain graphs labelled using matrix-element combination as shown by Day & Hawthorne (2022 ▸, Appendix *G*). Other abbreviations as given in Table 1 ▸.

Chain graph	〈*T*–*T*〉 (Å)	R〈*T*–*T*〉 (Å)	R〈*T*⋯*T*〉_min_ (Å)	*T*⋯*T* < γ	Planarity	Acy/cy-poly	*n*_t_:*n*_g_	c/r/t	Observed
(2×**1**, 4×**2^1^**) **a**	50.001	50.001–50.002	85.802–86.182	–	p	cy-3	1:2	r	No
(2×**1**, 2×**2^1^**, 2×**2^2^**) **a**	50.002	50.001–50.003	68.859–71.883	–	np	cy-3,5	1:2	r	No
(4×**1**, 3×**2**)	50.002	50.001–50.003	68.770–70.704	–	p	cy-4	1:1	r	No
(2×**1**, 4×**2^1^**) **b**	50.001	48.503–51.722	68.687–69.751	–	p	cy-3,4	1:2	r	No
(4×**1**, 1×**2**, 2×**2^1^**) **a**	50.004	50.001–50.016	62.530–65.713	–	np	cy-3,4 (s)	1:7	r	No
(4×**1**, 1×**2**, 2×**2^2^**) **b**	50.002	48.432–51.148	57.126–62.114	2	np	cy-3,5	1:2	r	No
(2×**1**, 4×**2^2^**) **b**	50.004	50.000–50.006	57.509–61.350	8	np	cy-4,6	1:5	t	No
(2×**1**, 4×**2^1^**) **c**	50.009	48.507–51.661	57.178–61.322	2	np	cy-4.5	1:9	t	No
(4×**1**, 1×**2**, 2×**2^1^**) **c**	50.004	49.958–50.316	53.187–58.132	2	np	cy-5	1:4	t	No
(4×**1**, 1×**2**, 2×**2^2^**) **c**	50.003	49.787–50.229	53.129–57.504	2	np	cy-4,6	1:3	t	No
(2×**1**, 2×**2**, 2×**2^1^**)	50.017	48.261–51.757	53.321–55.874	2	np	cy-3,5	1:2	r	No
(2×**1**, 2×**2**, 2×**2^2^**)	50.003	49.626–51.153	54.530–55.726	2	np	cy-4,6	1:2	t	No
(2×**1**, 2×**2^1^**, 2×**2^2^**) **b**	50.002	48.440–52.065	52.261–55.040	1	np	cy-3,7	1:7	t	No
(4×**1**, 1×**2**, 2×**2^1^**) **b**	50.003	49.347–50.340	51.228–52.070	1	np	cy-5	1:6	t	No

**Table 4:** Compatibility parameters for chain graphs with *e*/*n* = 2.00 Initial *T*–*T* distance (spring length) set to 50 Å, drag coefficient = 0.02, *k* = 0.0035–0.0052, *K* = −0.003, time step = 20 ms, cooldown time = 5 × 10^6^ ms. Theta (*q*_c_) for Barnes–Hut simulation was varied depending on complexity of the input graph. Values calculated after cooldown time has elapsed or until *T*⋯*T* separations have converged. Allowed variation in *T*–*T* = 47.55–52.45 Å, minimum allowable *T*⋯*T* = γ = 58.00 Å. Chain graphs labelled using matrix-element combination as shown by Day & Hawthorne (2022 ▸, Appendix *G*). Other abbreviations as given in Table 1 ▸.

Chain graph (*^c^V_r_*)	〈*T*–*T*〉 (Å)	R〈*T*–*T*〉 (Å)	R〈*T*⋯*T*〉_min_ (Å)	*T*⋯*T* < γ	Planarity	Acy/cy-poly	*n*_t_:*n*_g_	c/r/t	Observed
(1) ^4^ *V*_2_ (2×**2**, 2×**2^1^**)	50.012	50.004–50.022	86.124–86.616	–	p	cy-3	1:1	r	No
(2) ^4^ *V*_3_ (6×**1**, 3×**2**) **a**	50.006	50.003–50.010	70.654–70.718	–	np	cy-3,4	1:1	t	No
(3) ^4^ *V*_5_	50.008	49.999–50.027	53.956–55.331	3	np	cy-3,4	–	r	No
(4) ^4^ *V*_3_ (2×**2**, 4×**2^1^**)	50.011	50.001–50.027	50.291–54.315	2	np	cy-3,4	1:2	r	No
(5) ^4^ *V*_8_	51.309	49.028–53.894	43.774–45.703	8	np	cy-3,4	1:2	t	No
(6) ^4^ *V*_2_	50.003	50.001–50.003	42.263–42.789	2	np	cy-4	1:2	t	No
(7) ^4^ *V*_3_ (2×**2**, 4×**2^2^**)	50.005	50.002–50.010	27.870–30.030	4	np	cy-4	1:2	t	No
